# Niclosamide reverses adipocyte induced epithelial-mesenchymal transition in breast cancer cells via suppression of the interleukin-6/STAT3 signalling axis

**DOI:** 10.1038/s41598-019-47707-2

**Published:** 2019-08-05

**Authors:** Jones Gyamfi, Yun-Hee Lee, Byung Soh Min, Junjeong Choi

**Affiliations:** 1 College of Pharmacy, Yonsei Institute of Pharmaceutical Sciences, Yonsei University, Incheon, Republic of Korea; 2 College of Pharmacy and Research Institute of Pharmaceutical Sciences, Seoul National University, Seoul, Republic of Korea; 3 Department of Surgery, Yonsei University College of Medicine, 50 Yonsei-ro, Seodaemun-gu, Seoul, Republic of Korea

**Keywords:** Breast cancer, Molecular medicine

## Abstract

The microenvironment of breast cancer comprises predominantly of adipocytes. Adipocytes drive cancer progression through the secretion adipocytokines. Adipocytes induce epithelial mesenchymal transition of breast cancer cells through paracrine IL-6/Stat3 signalling. Treatment approaches that can target adipocytes in the microenvironment and abrogate paracrine signals that drive breast cancer growth and metastasis are urgently needed. Repositioning of old drugs has become an effective approach for discovering new cancer drugs. In this study, niclosamide, an FDA approved anthelminthic drug was evaluated for its anti-breast cancer activity and its ability to inhibit adipocytes induced EMT. Niclosamide potently inhibited proliferation, migration and invasion at low concentration and induced significant apoptosis at high concentrations in human breast cancer cell lines MDA-MB-468 and MCF-7. Additionally, niclosamide reversed adipocyte-induced EMT with a correlated inhibition of IL-6/Stat3 activation and downregulation of EMT-TFs TWIST and SNAIL. Moreover, niclosamide markedly impaired MDA-MB-468 and MCF-7 migration and invasion. We further found that the inhibitory effects of niclosamide on MDA-MB-468 and MCF-7 motility was closely related to destabilization of focal adhesion complex formation. With decreased co-localization of focal adhesion kinase (FAK) and phosphorylated paxillin (pPAX). Collectively, these results demonstrate that niclosamide could be used to inhibit adipocyte-induced breast cancer growth and metastasis.

## Introduction

The tumour microenvironment comprises proliferating tumour cells, the tumour stroma, infiltrating inflammatory cells and a variety of cancer associated cells^1,2^. The role of the tumour microenvironment in tumour progression has recently gained enormous attention, with studies reporting heterogeneous interactions between tumour cells and tumour stroma cells driving tumour progression^3–5^. In various tumours, the tumour microenvironment evolves into a unique environment suitable to promote the growth of the primary tumour^5^. The breast cancer microenvironment is unique since the breast tissue within which the tumour originates comprises predominantly of adipocyte. This makes adipocytes a unique component in the breast cancer microenvironment^6–8^. Adipocytes secrete various growth factors and cytokines collectively referred to as adipocytokines^9^. Leptin, adiponectin, autotaxin, interleukin 6 (IL-6), and transforming growth factor-β (TGF-β) have all been identified to be secreted by cancer-associated adipocytes^10^. These factors independently play diverse roles in tumour progression. Particularly with the studies reporting TGF-β and IL-6 secreted by adipocytes capable of inducing epithelial mesenchymal transition (EMT) in epithelial tumours^11–14^.

During tumour progression induction of EMT occurs via paracrine signals from cell in the tumour microenvironment, allowing tumour cells to infiltrate surrounding tissue and metastasize to distant sites^11,15^. Several growth factors, including TGF-β, WNT and IL-6 have been shown to trigger EMT in transformed cell lines^16,17^. These growth factors activate signalling pathways that activate downstream transcription factors, such as Snail and Twist^16,17^. A thorough understanding of the molecular mechanism by which growth factors induce EMT may help in the development of agents that block EMT-inducing signals from the tumour microenvironment or to inhibit critical down- stream signal transduction pathways. In breast cancer, studies have begun to identify the mechanisms by which adipocyte-induced EMT occurs, among the identified pathways are the TGF-β/Smad axis, TGF-β/STAT3 axis and the IL-6/STAT3 axis^8,11,13,18,19^. Increased secretion of IL-6 in the tumour microenvironment, promotes tumourigenesis by activation of the signal transducer and activator of transcription 3 (STAT3) and expression of its target genes involved with apoptosis, survival, proliferation, angiogenesis, invasiveness and metastasis^13,20^. Phosphorylated STAT3 (pSTAT3) localises to the nucleus and drives the expression of genes (TWIST and SNAIL) that regulate EMT^21,22^. Thus, the potential of blocking IL-6 and STAT3 activity has emerged as a potential therapeutic strategy^23–25^. Aberrant tyrosine phosphorylation of Stat3 has been detected at high frequency in diverse human cancer cell lines and tissues^26,27^. Various attempts have been made to develop agents that prevent STAT3 phosphorylation and/or dimerization and act as anti-cancer agent^27–31^. Repositioning of old drugs to treat cancers have emerged as a rapid means of developing anticancer agents^32,33^. Niclosamide is an orally bioavailable chlorinated salicylanilide, approved as an anthelmintic drug. Niclosamide has recently been reported to have antineoplastic activity targeting multiple intracellular signalling pathways including STAT3^34^. High-throughput screen identified niclosamide as potent Stat3 inhibitor, that suppresses Stat3 phosphorylation at Tyr705 in adrenocortical carcinoma and prostate cancer^35–37^. The potential of niclosamide for anti-cancer therapy is currently being evaluated in clinical trials in prostate and colorectal cancers^38,39^. However, the potential role of niclosamide on breast cancer cells, breast cancer microenvironment, breast tumour metastasis and its related molecular mechanism have not been completely investigated.

We have previously reported human adipocytes induce EMT in MDA-MB-468 and MCF-7 breast cancer cells through paracrine IL-6 activation of STAT3, enhancing breast cancer cell migration and invasion, by induction of EMT^19^. To determine if activated STAT3 activity can be targeted to limit the effects of adipocytes on breast cancer cells. We selected niclosamide based on available evidence of it potential to inhibit STAT3 phosphorylation and nuclear localization and systematically investigated its effects on adipocytes-induced EMT in MDA-MB-468 and MCF-7 breast cancer cells. We also explore the mechanism by which niclosamide may affect breast cancer cell behaviour. We found that adipocyte conditioned media can induce EMT is a manner comparable to differentiated adipocytes. Niclosamide inhibited adipocyte-induced effects and significantly decreased cell motility in a dose-dependent manner. Using stable STAT3 reporter cells, we showed that niclosamide significantly reduced STAT3 activity in both cells and inhibit breast cancer cell motility by destabilizing focal adhesion complex formation.

## Results

### Niclosamide inhibits human adipocyte differentiation

To determine if niclosamide can reverse/inhibit adipocyte induced EMT in breast cancer cells, we initially determine the direct effect of niclosamide on differentiation of human adipocytes. We treated preadipocytes with different doses of niclosamide (0, 0.125, 0.250, 0.500 and 1.00 µM) at day 0 of differentiation induction (Fig. S1a) and after day 3 of differentiation induction (Fig. S1d). Treatment with niclosamide concentration above 1.000 µM inhibited adipocyte differentiation resulting in cells detaching. Treatment with niclosamide either from day 0 or after day 3 of induction resulted in reduced adipocyte differentiation with significant reduction in the number and size of lipid droplets, with a correlated decrease in lipid accumulation (Fig. S1a,b,d,e). Triglyceride content of adipocytes differentiated in niclosamide decreased significantly in a dose dependent manner (Fig. S1c,f). These results indicate the ability of niclosamide of inhibit adipocyte differentiation in a dose dependent manner.

We next determined the effect of niclosamide (0, 0.125, 0.250, 0.500 and 1.00 µM) on fully differentiated human adipocytes. Treatment at concentration greater than 1.0 µM resulted in significant cell death. Low concentration of niclosamide (≤0.250 µM) resulted in gradual delipidation, deceased lipid accumulation and detachment of adipocytes over 48 hours (Fig. S1g,h). Triglyceride levels of differentiated adipocytes decreased after treatment with niclosamide (Fig. S1i). Glycerol is a major by-product of triglyceride hydrolysis; we determine levels of glycerol in adipocyte media after treatment with niclosamide. Glycerol release after niclosamide treatment increased in a concentration and time-dependent manner (Fig. S1j). These results indicate that in fully differentiated adipocytes, niclosamide induces cell death at high concentration (>0.750 µM) and at low concentration (0.250 µM) niclosamide results in significant delipidation from increased hydrolysis of triglycerides as shown by decrease in triglyceride content and an increase in glycerol release (Fig. S1i,j). We also assessed how reduced differentiation of adipocytes in the presence of niclosamide affect breast cancer cell proliferation and viability (Fig. S2). Breast cancer cells cultured with niclosamide differentiated adipocyte CM, had decreased proliferation and viability in a dose-dependent manner (0.125, 0.250, 0.500 and 1.00 µM). Breast cancer cells treated with 0.500 µM and 1.000 µM niclosamide differentiated adipocyte CM had significantly reduced proliferation and reduced viability (Fig. S2). These finding indicate that the differentiation status of adipocyte my affect their capacity to influence breast cancer cell behaviour as has been previously reported^40^.

Collectively, these results indicate that niclosamide can inhibit adipocyte differentiation and induce triglyceride hydrolysis in differentiated adipocytes. The effect of niclosamide on adipocyte differentiation also affects the ability of conditioned media from niclosamide differentiated adipocytes to enhance proliferation and maintain viability of breast cancer cells.

### Niclosamide induces apoptosis in breast cancer cells

**Figure 1:**
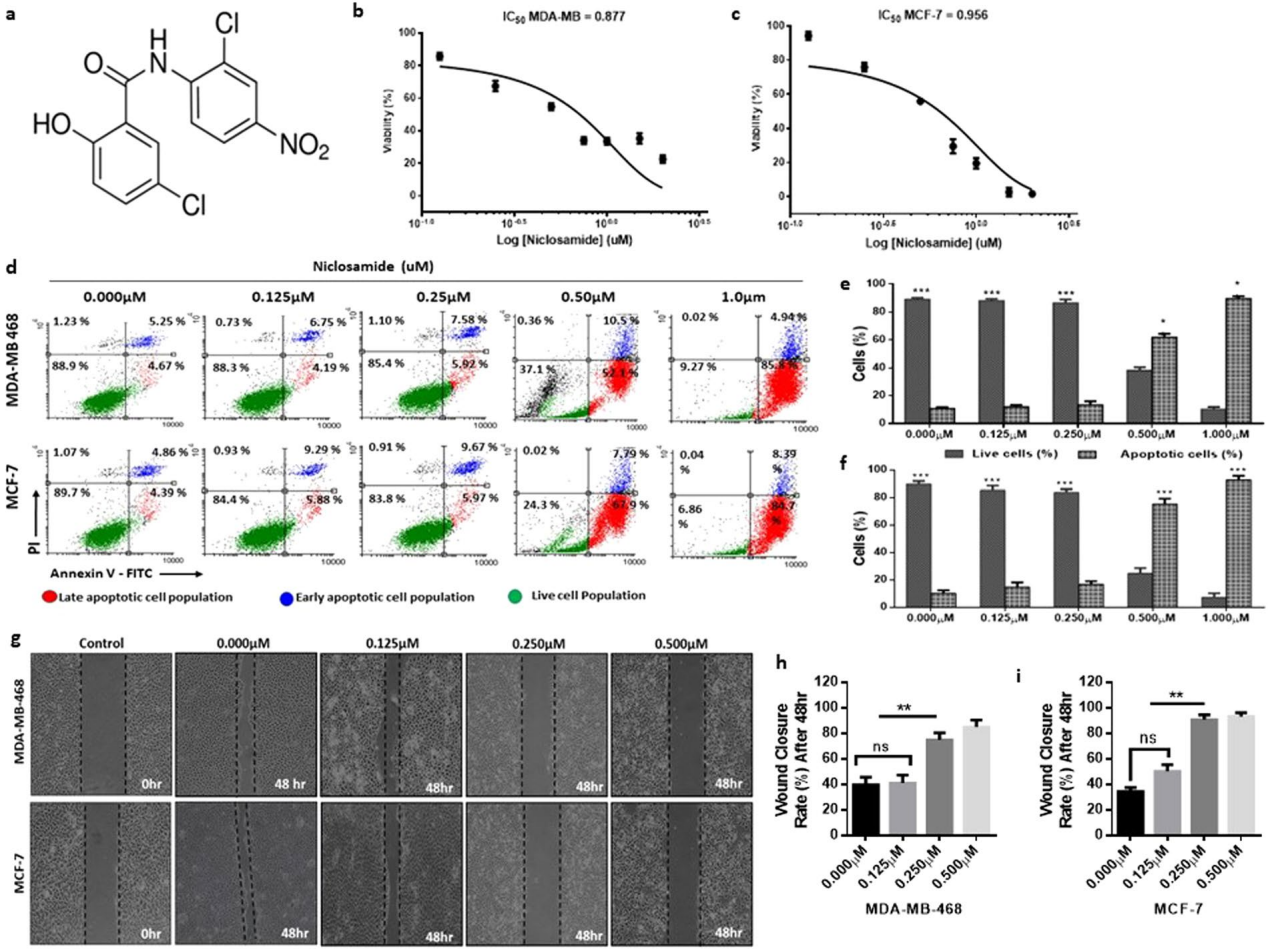
Niclosamide induces apoptosis in breast cancer cells. **(a)** Structure of niclosamide **(b)** Cell viability curves for effect of niclosamide on breast cancer cells (MDA-MB-468 and MCF-7) following 24, 48 and 72-hour exposure. **(c–e)** Evaluation of apoptosis in breast cancer cells treated with niclosamide using Annexin V/PI dual-labelling technique (**c)**. Representative images of flow cytometry analysis of apoptosis (**d)**. Quantitative analysis of percentage of MDA-MB-468 cells undergoing apoptosis. (**e**) Quantitative analysis of percentage of MCF-7 cells undergoing apoptosis. **(f–h)** Evaluation of cell motility in breast cancer cells treated with different concentrations of niclosamide after 48 hours (0.000, 0.125, 0.250, 0.500, 1.000 µM) (**f)**. Representative images of wound healing assay (magnification, X100) (**g)**. quantitative analysis of wound closure rate (%) in MDA-MB-468 breast cancer cells (**h)**. quantitative analysis of wound closure rate (%) in MCF-7 breast cancer cells. All results are representative of 3 independent experiments. (Data indicate mean ± SD; ***p < 0.001; **p < 0.01; *p < 0.05).

To explore the direct effects of niclosamide on breast cancer cells, we treated MDA-MB-468 and MCF-7 cells with different concentration of niclosamide for 48 hours and determined its effect on cell viability. After 48 hours, the IC_50_ for niclosamide for MDA-MB-468 and MCF-7 cells were 0.877 µM and 0.956 µM respectively (Fig. 1b). Treatment of cancer cells with niclosamide is reported to decrease cell viability via the induction of apoptosis^41,42^. To investigate whether niclosamide can induce apoptosis in breast cancer cells we treated breast cancer cells with different concentrations of niclosamide for 24 hours and determined the proportion of live cells, early apoptotic and late apoptotic cells using Annexin V-FITC/PI dual labelling (Invitrogen). For MDA-MB-468 cells, treatment with niclosamide increased the number of cells in late apoptosis from 52.1% (with 0.500 µM) to 85.8% (with 1.00 µM), with a decrease in number of live cells from 37.1% to 9.27% (Fig. 1c,d). Similar results were obtained in MCF-7 cells with the number of cells in late apoptosis increasing from 67.9% (with 0.500 µM) to 84.7% (with 1.00 µM) and the number of live cells decreasing from 24.3% to 6.86% (Fig. 1c,e). In cells treated with 0.125 µM and 0.250 µM of niclosamide, the number of cells in apoptosis was 10.94% and 14.50% respectively for MDA-MB-468 cells (Fig. 1c,d) and 15.17% and 15.64% respectively for MCF-7 cells (Fig. 1c,e). At low concentrations niclosamide did not induce significant apoptosis, analysis of cell viability showed that cell viability in both MDA-MB-468 and MCF-7 decreased when treated with 0.250 µM (Fig. S3a,b). This dose dependent effect of niclosamide on breast cancer cell viability was also observed when cells where treated with different concentrations of niclosamide and cytotoxicity assessed at different points (Fig. S3c,d). Niclosamide has been reported to inhibit cancer cell migration and motility^35,42^, hence we investigated the effect of different concentrations of niclosamide on breast cancer cell motility by wound healing assay. In both MDA-MB-468 and MCF-7 cells, increasing the concentration of niclosamide resulted in a significant decrease in breast cancer cell motility (Fig. 1f–h). In cells treated with 0.500 µM niclosamide, detached and apoptotic cells were observed. Collectively, these results indicate a dose-dependent effect of niclosamide on MDA-MB-468 and MCF-7 cells, with high concentrations niclosamide inducing significant apoptosis and a significant decrease in cell viability. At low concentrations, niclosamide does not significantly decrease cell viability but decreases breast cancer cell motility.

### Niclosamide inhibits adipocyte induced breast cancer cell growth

**Figure 2:**
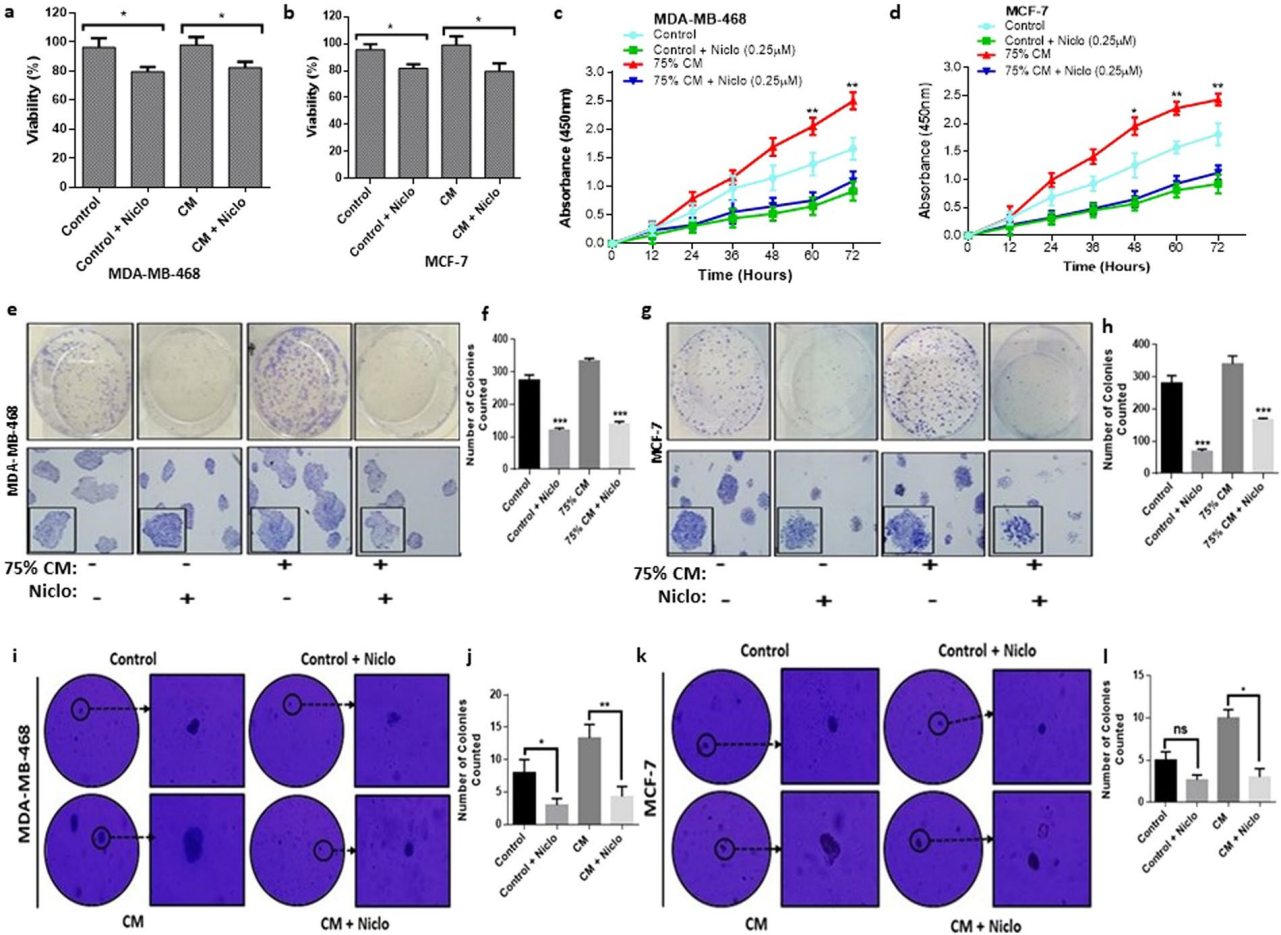
Effects of niclosamide on breast cancer cells growth. (**a,b**) Quantitative analysis of MDA-MB-468 and MCF-7 cells viability by CCK-8 after treatment with niclosamide (0.250 µM) and cultured for 48 h. **(c**,**d)** Proliferation curves of MDA-MB-468 and MCF-7 breast cancer cells cultured with/without adipocyte-CM and treated with/without (0.250 µM) and cultured for 48 hr. Cell proliferation rates were estimated using EZ-Cytox kit at specific times (12, 24, 36, 48, 60 and 72 hours). **(e–l)** Evaluation of the colony formation capabilities of breast cancer cells treated with niclosamide. (**e)**. Representative images of clonogenic formation ability of MDA-MB-468 (**f)**. Quantitative analysis of number of colonies formed by MDA-MB-468 (**g)**. Representative images of clonogenic formation ability of MCF-7 (**h)**. Quantitative analysis of the number of colonies formed by MCF-7. (**i**) Representative images of soft agar colony formation ability of MDA-MB-468 (**j)**. Quantitative analysis of the number of soft agar colonies formed by MDA-MB-468 (**k)**. Representative images of soft agar colony formation ability of MCF-7 (**l)**. Quantitative analysis of the number of soft agar colonies formed by MCF-7. All results are representative of 3 independent experiments. (Data indicate mean ± SD; ***p < 0.001; **p < 0.01; *p < 0.05).

Since, niclosamide induced adipocyte lipolysis, niclosamide treatment of directly co-cultured breast cancer cell and adipocytes to determine its effects was not feasible. Hence, to investigate the effect of niclosamide on adipocyte-breast cancer cell interaction, subsequent experiment with niclosamide was conducted with 75% adipocyte-conditioned media (CM) collected from fully differentiated human adipocytes (Fig. S3a–f). To investigate whether niclosamide can reduce breast cancer cells viability, MDA-MB-468 and MCF-7 cells were cultured in adipocyte CM with/without niclosamide (0.250 µM) for 48 hours and cell viability determined. In both MDA-MB-468 and MCF-7 cells adipocyte-conditioned media did not alter cell viability compared to control cells after 48 hours (Fig. 2a,b). However, the presence niclosamide decreased cell viability in both control and co-cultured cells in both MDA-MB-468 and MCF-7 cells. We determined the effects of niclosamide on MDA-MB-468 and MCF-7 proliferation. Treatment of MDA-MB-468 and MCF-7 cells with niclosamide for 24, 48 and 72 hours resulted in a decrease in cell proliferation rate in control cell and in cells cultured with adipocyte CM (Fig. 2c,d). In both cells, adipocyte CM significantly increased cell proliferation rate in a time-dependent manner but treatment with niclosamide significantly reversed adipocyte induced proliferation (Fig. 2c,d). Thus, although, adipocyte-CM increased proliferation rate it does not affect cell viability and treatment with niclosamide reverses adipocyte induced increased proliferation and decreases breast cancer cell viability. Combined with apoptosis results (Fig. 1a,b), these findings indicate that adipocyte-CM can enhance proliferation and treatment with niclosamide induce apoptosis resulting in a decrease in cell viability.

Furthermore, we determined the effect of adipocyte CM on the ability of breast cancer cells to form colonies and alter breast cancer anchorage-independent growth (the ability of transformed cells to grow and form colonies on semi-solid matrices). We found that in both breast cancer cell lines, adipocyte CM resulted in the formation of more and bigger colonies compared to control cells (Figs 2e–l and S4a–i). Colonies formed by MCF-7 were larger than MDA-MB-468 cells, although the number of colonies did not differ significantly between the two cells (Fig. 2e–l). Treatment with niclosamide significantly inhibited the colony formation abilities of both cells and resulted in the formation of smaller and inconspicuous colonies (Fig. 2e–l). Niclosamide exert a dosage-dependent effect on the ability of cells to form colonies (Fig. S4a,b) Together, these results indicate the ability of niclosamide to inhibit breast cancer cell growth by reducing their proliferation rate, viability and ability to form colonies *in-vitro*.

### Niclosamide inhibits adipocytes induced cancer cell migration and invasion

**Figure 3:**
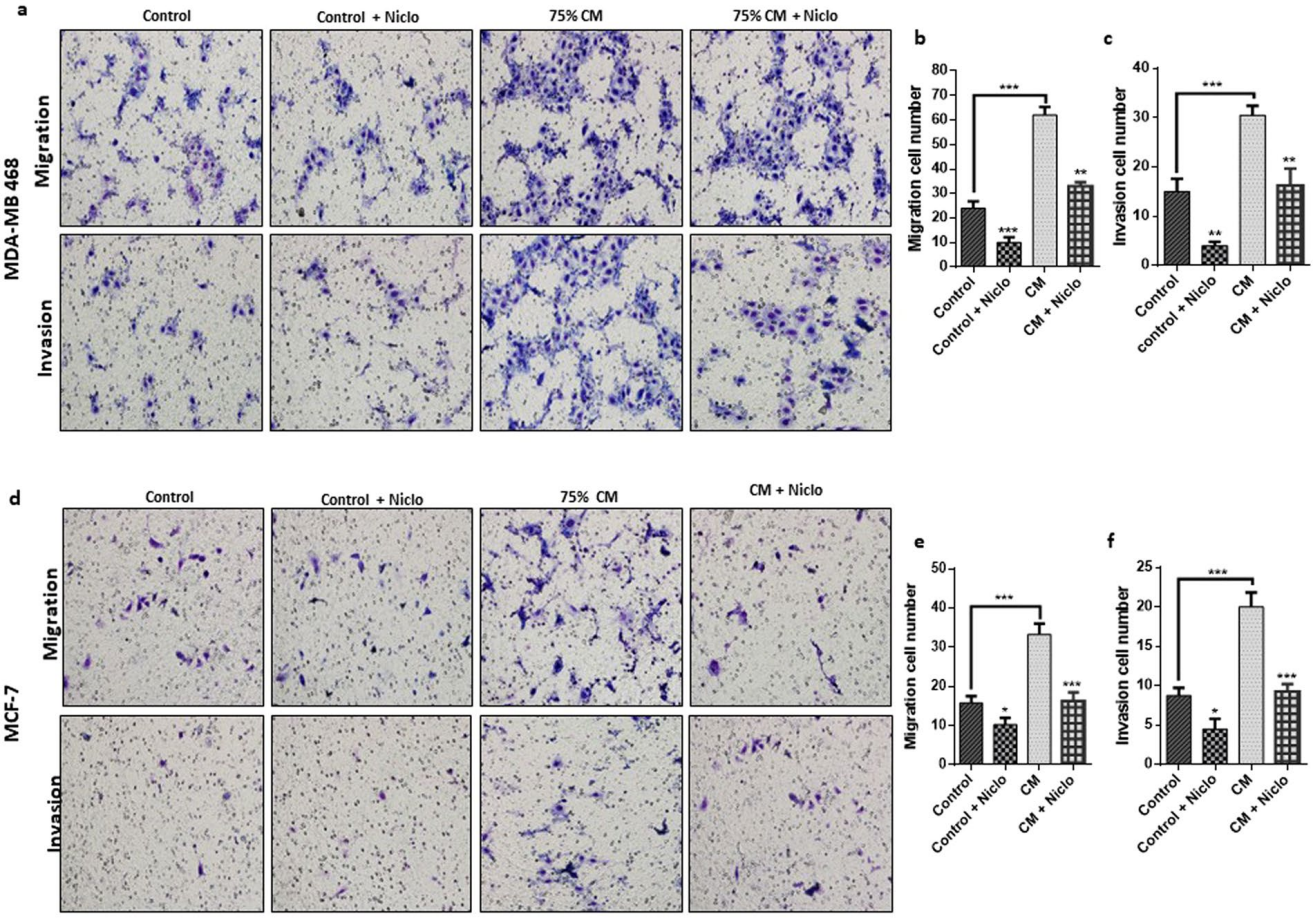
Niclosamide inhibits adipocyte-induced cell migration and invasion. (**a–f)** Evaluation of the effects of niclosamide on breast cancer cell migration and invasion by transwell assay with/without matrigel. **(a**,**b)** Representative images of MDA-MB-468 and MCF-7 migration and invasion (x200 magnification) cultured with/without adipocyte-CM and treated with/without niclosamide (0.250 µM). **(c–f)** Quantitative analysis of migration and invasion cell numbers of MDA-MB-468 and MCF-7 breast cancer cells cultured with/without adipocyte-CM and treated with/without niclosamide (0.250 µM). All results are representative of 3 independent experiments. (Data indicate mean ± SD; ***p < 0.001; **p < 0.01; *p < 0.05).

Studies have reported severally the ability of adipocytes to promote cancer cell migration and invasion^11^. To investigate if niclosamide can antagonize this effect, we investigated the migration and invasion capabilities of MDA-MB-468 and MCF-7 cells cultured in adipocyte CM with/without niclosamide using the transwell assay with or without matrigel coating. Comparing adipocyte CM treated cells to control cells the migration and invasion capabilities were indeed enhanced in both cells (Fig. 3a–f). We found that niclosamide inhibited the migration and invasion of both MDA-MB-468 and MCF-7 cells (Fig. 3a–f). In cells cultured with adipocyte CM, niclosamide inhibited migration and invasion but the number of migrated and invasion cells was higher than control cells treated with niclosamide (Fig. 3a–f). Collectively, these results show that niclosamide is capable of inhibiting adipocyte-induced migration and invasion in both breast cancer cell lines.

### Niclosamide reverses adipocytes induced EMT in breast cancer cells

**Figure 4:**
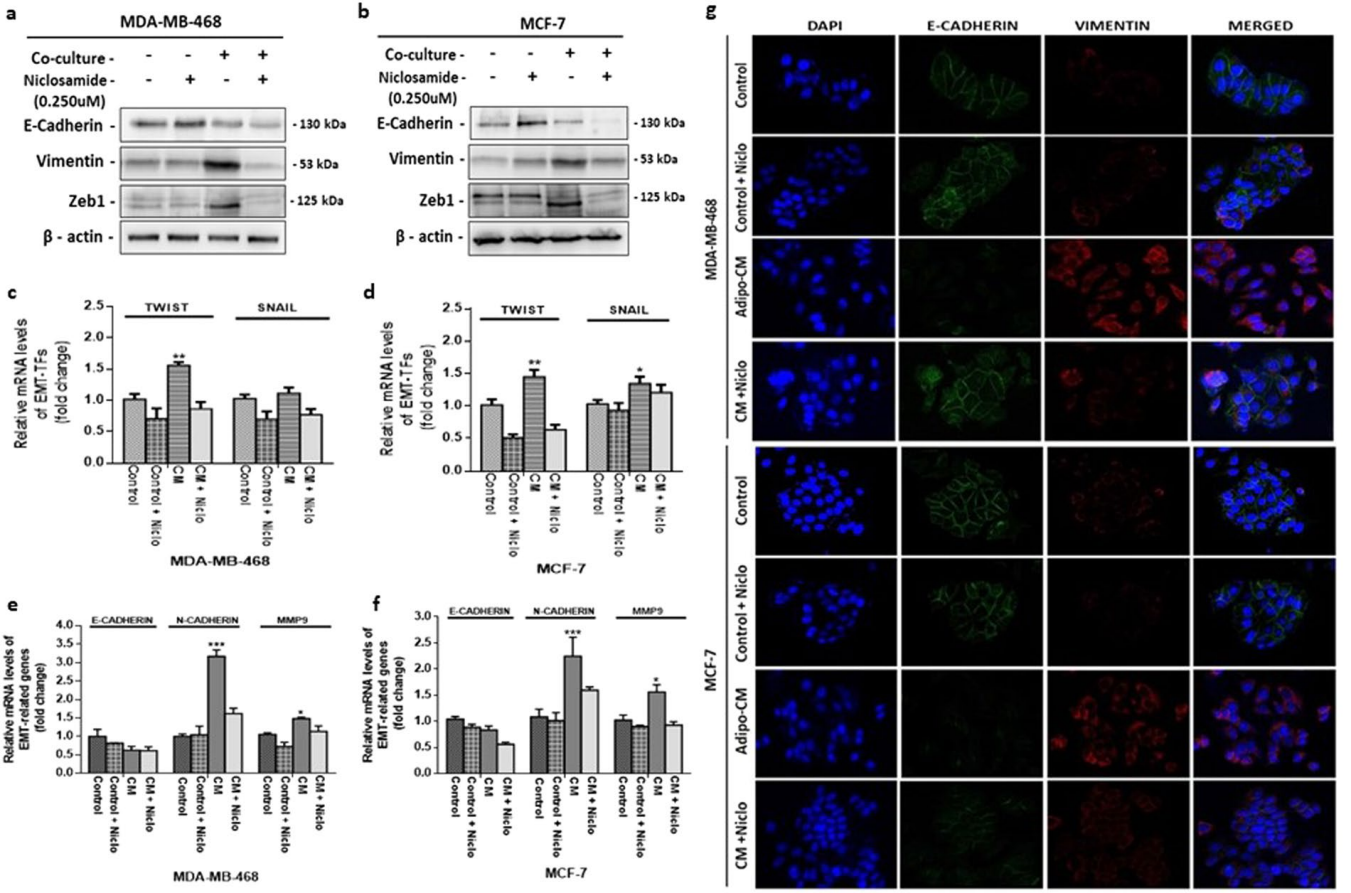
Niclosamide reverses adipocyte induced EMT. (**a,b**) Evaluation for the emergence of EMT phenotype in MDA-MB-468 and MCF-7 breast cancer cells treated with niclosamide. Western blot images for E-cadherin, Vimentin and ZEB1. Full-length blots are presented in Supplementary Fig. 10. **(c**,**d)** Quantitative PCR (qt-PCR) comparing the expression of EMT-TFs (TWIST and SNAIL) in MDA-MB-468 and MCF-7 breast cancer cells cultured with/without adipocyte-CM and treated with/without niclosamide. **(e**,**f)** Qt-PCR comparing the expression of EMT-related genes (MMP9, N-cadherin and E-cadherin) in MDA-MB-468 and MCF-7 breast cancer cells cultured with/without adipocyte-CM and treated with/without niclosamide. Relative mRNA expression was normalized to GAPDH. All results are representative of 3 independent experiments. (**g**) Immunofluorescence staining of breast cancer cells cultured with/without adipocyte-CM and treated with/without niclosamide, E-cadherin (epithelial marker) co-stained with Vimentin (mesenchymal marker) and DAPI (staining of nuclei) shown at 200-fold magnification. All results are representative of 3 independent experiments. (Data indicate mean ± SEM; ***p < 0,001; **p < 0.01; *p < 0.05).

We recently reported that human adipocytes induced EMT occur via the IL-6/STAT3 axis in breast cancer cells^19^. Niclosamide is reported as potent repressor of STAT3 activity, hence, we investigated if niclosamide can counteract adipocyte-induced EMT. MDA-MB-468 and MCF-7 were cultured with adipocyte-CM and treated with niclosamide for 48 hours followed by western blot assay to determine the expression of levels of markers involved with EMT. Adipocyte-CM treated breast cancer cells upregulated the levels of mesenchymal markers (Vimentin and Zeb1) and downregulated epithelial marker (E-cadherin) (Figs 4a,b and S5a) indicating that adipocyte CM can promote EMT. Treatment with niclosamide counteracted adipocyte-induced EMT in both cells, upregulating E-cadherin and downregulating vimentin and Zeb1 (Figs 4a,b and S5a). We further substantiated these results by determining the mRNA levels of two EMT-associated TFs proposed to be regulated by STAT3 (TWIST and SNAIL). Adipocytes CM treated MDA-MB-468 and MCF-7 cells upregulated the levels of TWIST and SNAIL, compared to control cells (Fig. 4c,d) and in cells treated with niclosamide TWIST and SNAIL expression was downregulated (Fig. 4c,d). We also examined the mRNA levels of markers associated with EMT (E-cadherin, N-cadherin and MMP9), consistent with characteristics of cells in EMT, adipocyte CM downregulated E-cadherin with upregulation of N-cadherin and MMP9 in both MDA-MB-468 and MCF-7 (Fig. 4e,f). Immunofluorescence images of EMT markers e-cadherin and vimentin was consisted with other findings (Figs 4g and S7). Together, these results indicate that niclosamide significantly reverses adipocyte-induced EMT is MDA-MB-468 and MCF-7 cells, decreasing the expression of EMT-TFs TWIST and SNAIL.

### Niclosamide represses adipocyte-induced IL-6 expression and STAT3 activation

**Figure 5:**
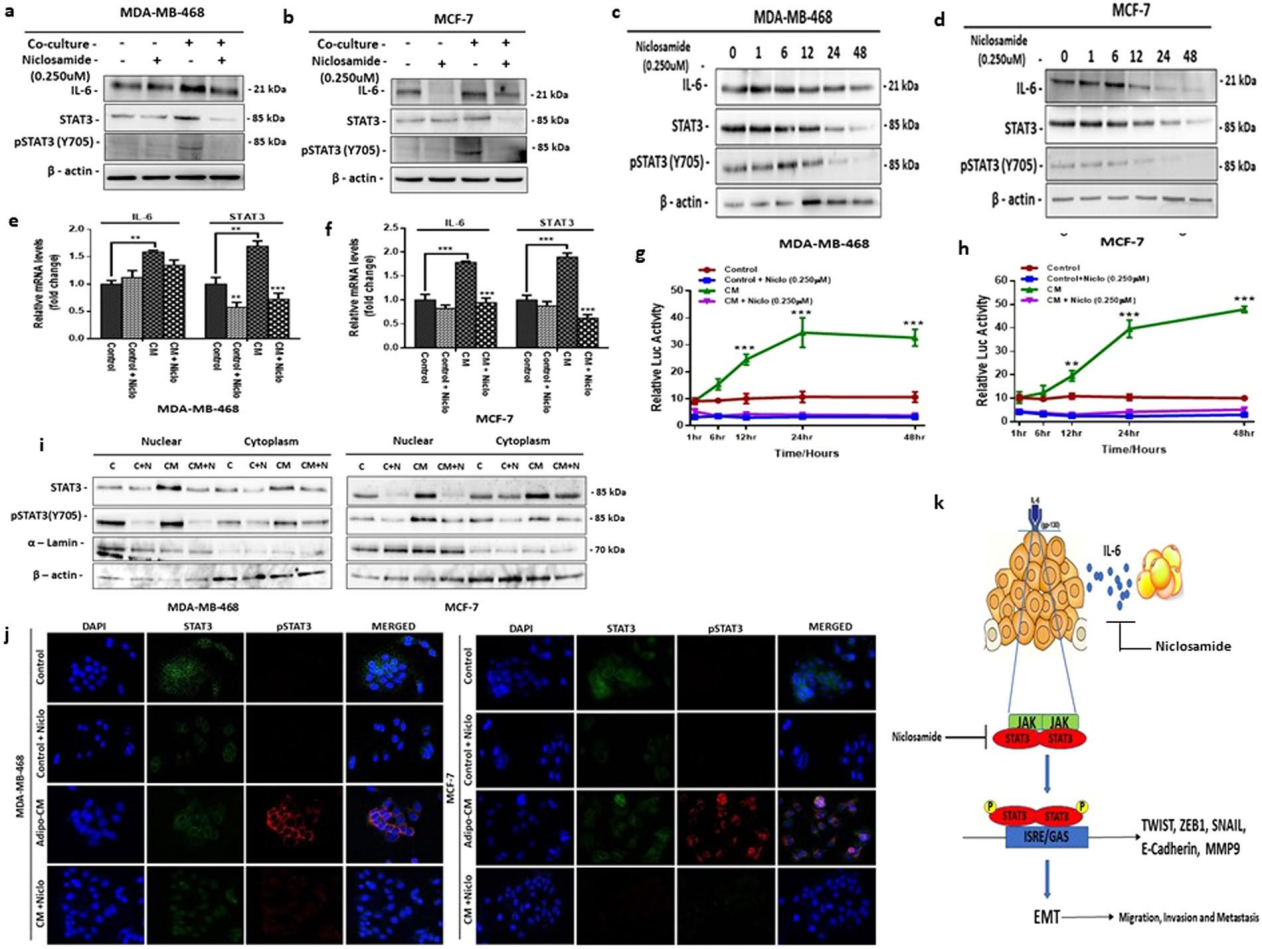
Niclosamide attenuates adipocyte-induced IL-6 expression and STAT3 activation. (**a**,**b)** Evaluation of the effect of niclosamide on the IL-6/STAT3 signalling axis in MDA-MB-468 and MCF-7 cells. Western blot images for IL-6, STAT3 and pSTAT3. **(c**,**d)** Western blot analysis for the expression levels of IL-6, STAT3 and pSTAT3 at different time points (0, 1, 6, 12, 24 and 48 h) after co-culture with adipocyte CM followed by treatment with 0.250 µM niclosamide for 48 hours. Full-length blots are presented in Supplementary Fig. 10. **(e**,**f)** Quantitative PCR (qt-PCR) comparing the expression of IL-6 and STAT3 in MDA-MB-468 and MCF-7 breast cancer cells cultured with/without adipocyte-CM and treated with/without niclosamide (0.250 µM). Relative mRNA expression was normalized to GAPDH. **(g**,**h)** Relative luciferase activity in MDA-MB-468 and MCF-7 cells transfected with STAT3 luciferase reporter plasmid and cultured with/without niclosamide Luciferase activity was normalized against a non-inducible luciferase construct. (**i**) Total nuclear and cytoplasmic levels of P-STAT3 and total STAT3 in MDA-MB-468 and MCF-7 breast cancer cells treated with niclosamide for 48 h was analysed by western blot, α-lamin and β-actin were used as loading controls for nuclear and cytoplasmic fraction respectively (**j)**. Immunofluorescence staining of breast cancer cells treated with niclosamide, STAT3 co-stained with pSTAT3 and DAPI (staining of nuclei) shown at 200-fold magnification. (**i**) Schematic representation of niclosamide effects on the interaction between adipocytes and breast cancer cells. Adipocytes secrete IL-6 which binds to the IL-6R on breast cancer cells, resulting in STAT3 phosphorylation and nuclear location. Nuclear localised STAT3 drives the expression of transcription factors (TWIST and SNAIL) and that enhance EMT. Niclosamide inhibit adipocyte-induced EMT by inhibiting STAT3 phosphorylation and nuclear localisation. All results are representative of 3 independent experiments. (Data indicate mean ± SEM; ***p < 0,001; **p < 0.01; *p < 0.05).

In breast cancer cells, IL-6 expression activates STAT3 which subsequently upregulate the expression of IL-6 creating an IL-6/STAT3 autocrine loop allowing for continual activation of the axis to drive tumour growth^26^. Hence, we investigated if treatment with niclosamide can downregulate the IL- 6/STAT3 axis and reverse adipocyte-induced EMT. Western blot assays showed that adipocyte upregulated IL-6 expression in both MDA-MB-468 and MCF-7 cells and niclosamide is capable of repressing adipocyte-induced IL-6 expression (Figs 5a,b and S5b). Analysis of IL-6 expression  levels by western blot in breast cancer cells after culturing in adipocyte CM for 48 hours followed by treatment with niclosamide shows a steady decrease in IL-6 levels at regular time levels (Fig. 5c,d). In both MDA-MB-468 and MCF-7, expression of IL-6 was high after culturing in adipocyte CM and decreased steadily over the next 48 hours after treatment with niclosamide (Fig. 5c,d). Quantitative PCR for IL-6 mRNA expression was consistent with western blots results (Fig. 5e,f), in both MDA-MB-468 and MCF-7 cells culturing with adipocyte CM increased IL-6 mRNA expression and treatment with niclosamide significantly decreased IL-6 mRNA expression (Fig. 5e,f).

To investigate the effect of niclosamide on STAT3 activity, we generated stable luciferase STAT3 reporting MDA-MB-468 and MCF-7 cells, and subsequently cultured STAT3 stable cells with adipocyte CM with/without niclosamide and monitored STAT3 luciferase activity at regular time intervals over 48 hours. Our results showed that in both MDA-MB-468 and MCF-7, treatment with adipocyte CM gradually increased STAT3 activity for the first 24 hours and remained consistently active over the 48 hours’ period (Fig. 5g,h) whereas in control cells, STAT3 activity remained stable over the period (Fig. 5g,h). In niclosamide treated, control and adipocyte CM cells, STAT3 activity was deceased significantly and remained low over the period (Fig. 5g,h). Results from time series analysis of STAT3 expression after treatment with niclosamide by western blot assay was consistent with STAT3 luciferase assay findings (Fig. 5c,d**)**. Quantitative PCR for STAT3 mRNA expression in both MDA-MB-468 and MCF-7 showed a significant increase in cells cultured with adipocyte CM and treatment with niclosamide significantly decreased STAT3 mRNA expression (Fig. 5e,f). Furthermore, our results show that adipocyte is capable of activating STAT3 in breast cancer cells evidenced by increased expression of phosphorylated STAT3 (pSTAT3, Y705). Western blot assay comparing cells cultured in adipocyte CM to control cells, showed that pSTAT3 expression increased in cell cultured in adipocyte CM (Figs 5a,b and S5b), and in cells cultured with niclosamide, pSTAT3 expression was not seen (Fig. 5a,b). Analysis of pSTAT3 expression levels by western blot in breast cancer cells after culturing for 48 hours followed by treatment with niclosamide shows a steady decrease in pSTAT3 expression at regular time levels (Fig. 5c,d).

STAT3 regulation of gene expression occurs via phosphorylation and nuclear localization of STAT3 allowing it to bind to key targets. Since adipocyte induces a constant activation of STAT3 (pSTAT3) we examined the localization of pSTAT3 in adipocyte CM treated cells with/without niclosamide. Western blot assay of cytoplasmic and nuclear fraction of cells cultured with adipocyte CM showed a significant nuclear localization of pSTAT3 in both MDA-MB-468 and MCF-7 cells (Fig. 5i). Niclosamide treatment inhibited pSTAT3 phosphorylation and decreased it nuclear localization (Fig. 5i). Increased expression and nuclear localization of pSTAT3 was confirmed my immunofluorescence. Results showed a strong nuclear localization of pSTAT3 in cells cultured with adipocyte CM (Figs 5j and S6). Images of cells cultured with niclosamide did not show STAT3 and pSTAT3 expression (Figs 5j and S7). Altogether, these results indicate that niclosamide can inhibit STAT3 activation and nuclear localization. Thus, niclosamide is a potent inhibitor of adipocyte-induced EMT in breast cancer cell by inhibiting the IL-6/STAT3 axis in breast cancer cells **(**Fig. 5k**)**.

### Niclosamide impairs focal adhesion complex formation and inhibits breast cancer cell motility

**Figure 6:**
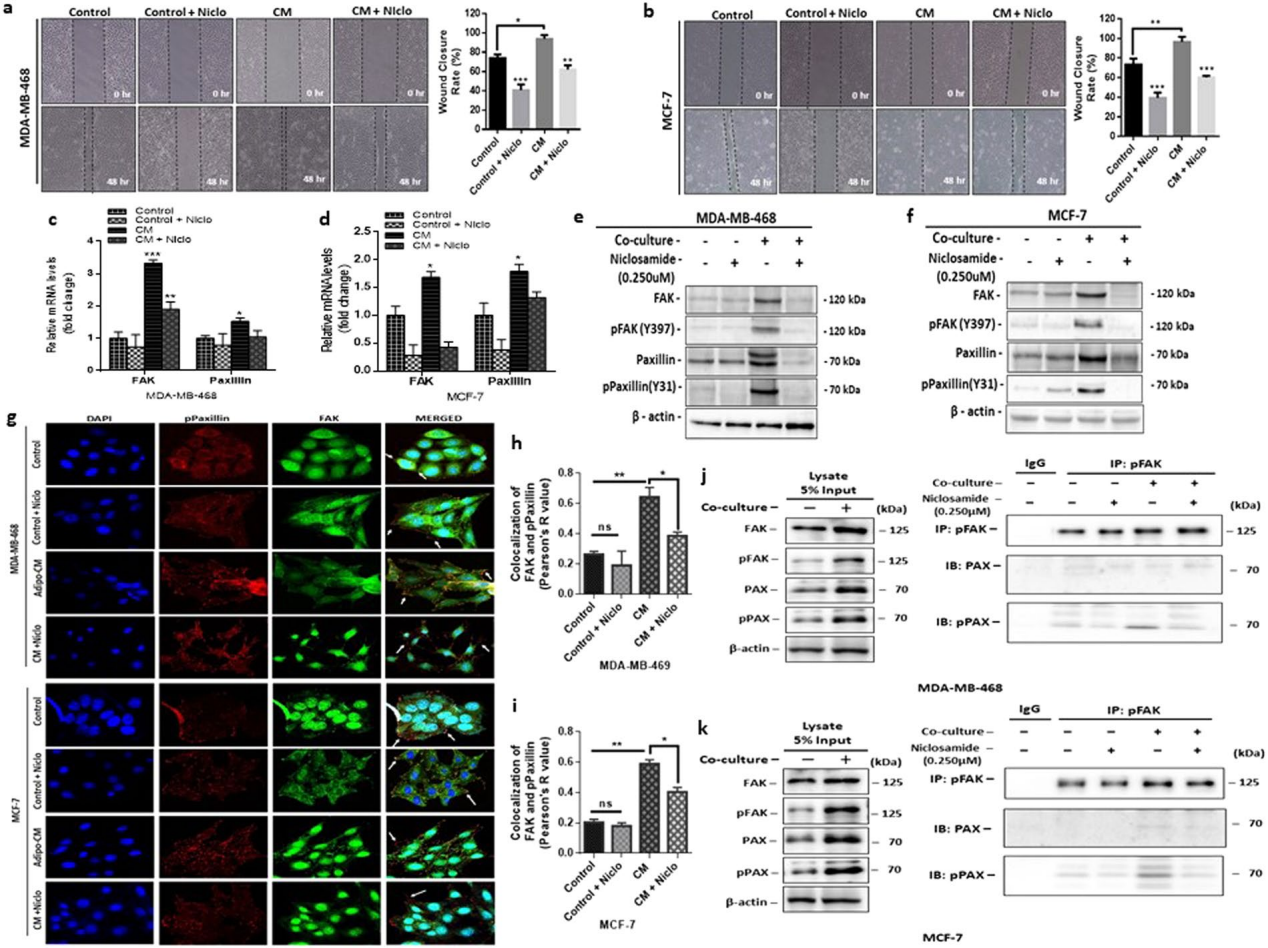
Niclosamide destabilizes focal adhesion complex formation. (**a**,**b)** Representative images of wound healing assay (magnification, x100) and quantitative analysis of wound closure rate (%) in MDA-MB-468 and in MCF-7 cells treated with niclosamide (0.250 µM). **(c**,**d)** Quantitative PCR (qt-PCR) comparing the expression of FAK and paxillin in MDA-MB-468 and MCF-7 breast cancer cells cultured with/without adipocyte-CM and treated with/without niclosamide (0.250 µM). Relative mRNA expression was normalized to GAPDH. **(e**,**f)** Evaluation of the effect of niclosamide on the focal adhesion complex formation in MDA-MB-468 and MCF-7 cells. Western blot images for FAK, pFAK (Y397), Paxillin, and pPaxillin (Y31). B-actin was used as loading control. Full-length blots are presented in Supplementary Fig. 10. **(g)** Immunofluorescence staining of breast cancer cells cultured with/without adipocyte-CM and treated with/without niclosamide (0.250 µM), FAK co-stained with pPaxillin and DAPI (staining of nuclei) shown at 200-fold magnification. **(h**,**i)** Pearson’s co-localization co-efficient for FAK and pPaxillin in MDA-MB-468 and MCF-7 cells cultured with adipocyte CM with/ without 0.250 µM niclosamide. **(j**,**k)** pFAK interacts with phosphor-paxillin and mediates cancer cell motility in co-cultured cells. Coimmunoprecipitation (CoIP) assay was performed to examine the interaction between pFAK, Paxillin and pPaxillin in MDA-MB-468 and MCF-7 cells after co-culture with adipocyte CM with/without niclosamide. Niclosamide destabilizes the interaction between pFAK and pPaxillin and limits cancer cell motility. 5% of whole cell lysate was assayed for comparison. Western blot was probed with anti-paxillin and anti-phospho-paxillin. All results are representative of 3 independent experiments. (Data indicate mean ± SEM; ***p < 0,001; **p < 0.01; *p < 0.05).

Dysregulation of the focal adhesions (FAs) formation results in decreased cell motility and in breast cancer studies have shown that inhibition of focal adhesion kinase impairs invadopodia formation and breast cancer cell invasion^43^. Since low concentrations of niclosamide decreased breast cancer cell motility, we investigated the effect of adipocyte CM and niclosamide of FAs formation and dynamics. Wound healing assay showed an increased wound closure rate in both MDA-MB-468 and MCF-7 cells cultured with adipocyte CM (Fig. 6a,b). When cells are cultured in adipocyte CM with niclosamide wound closure rate decreased significantly (Fig. 6a,b).

We determined the mRNA expression levels focal adhesion kinase (FAK) and paxillin (PAX) by quantitative-PCR, FAK and PAX mRNA levels were significantly increased in cells cultured with adipocyte CM without niclosamide (Fig. 6c,d) compared to cells cultured with adipocyte CM with niclosamide (Fig. 6c,d**)**. These finding were confirmed by determining the protein expression levels of FAK, phosphor-FAK, paxillin and phospho-paxillin by western blot. Western blot assay showed that FAK and PAX expression increased significantly in breast cancer cells cultured with adipocyte CM, and treatment with niclosamide significantly decreased their expression (Figs 6e,f and S5b). Importantly, phosphorylated FAK (pFAK) phosphorylates key proteins in the FA complex such as paxillin and allow components of the focal adhesion complex to associate and bind to FAK. Thus, we determined the expression of phosphorylated FAK(Y397) and phosphorylated paxillin (pPAX, Y31) by western blots. Results showed that in both MDA-MB-468 and MCF-7 cells, levels of pFAK and pPAX were significantly higher in cells cultured with adipocyte CM than in control cells (Figs 6e,f and S5b) and cells cultured with adipocyte CM with niclosamide had a significant decrease in the expression of pFAk and pPAX (Figs 6e,f and S5b).

Using concurrent dual-colour imaging we assessed the co-localization of FAK and phosphorylated paxillin by immunofluorescence, our results revealed an increase FAK and pPAX co-localization in cells cultured with adipocyte CM compared with control cells (Fig. 6g–k). In cells cultured with niclosamide, cells appeared clustered together with indistinct complexes (Fig. 6g). To test the hypothesis that treatment with niclosamide destabilises the formation of focal adhesion complexes by reducing the interaction between pFAK and pPAX, co-immunoprecipitation assay was performed. In both MDA-MB-468 and MCF-7 cells, pFAK antibody co-immunoprecipitated higher levels of pPAX in cell treated with adipocyte CM, in cells cultured in adipocyte CM with niclosamide the amount of pPAX proteins immunoprecipitated was significantly decreased **(**Fig. 6j,k**)**. These data indicate that in the presence of adipocyte CM, breast cancer cells form stable focal adhesion by pFAK interacting with pPAX that allow for their migration and treatment with niclosamide reduces the interaction between pFAK and pPAX destabilising the focal adhesion and reducing cell motility. Taken together, these results indicate that niclosamide can impair focal adhesion formation by inhibiting phosphorylation of key components of the focal adhesion complex and reduces the interactions between components (pFAK and pPAX) of the focal adhesion complex, resulting in decreased cell motility.

## Discussion

The quest to find new anti-cancer agents have resulted in drug-repositioning of old drugs for cancer therapy. Drug repositioning offers a great advantage since their pharmacokinetics and toxicity profiling are already establish, this facilitates rapid translation of bench work to clinics^32,33^. Niclosamide a potent inhibitor of STAT3 signalling pathway has showed potential to be repositioned as an antineoplastic agent. Several studies have demonstrated the antitumour activity of niclosamide in various types of cancers (e.g., leukaemia, colorectal, prostate and breast)^34^ and clinical trials for niclosamide are on-going for prostate and colorectal cancers^38,39^. The underlying mechanism of action for niclosamide in different types of cancers seems to differ. With studies reporting niclosamide to inhibit NF-kB, mTOR and STAT3 pathway in uveal melanoma, cervical and prostate cancer respectively^35,44,45^. In line with these efforts, we evaluated the anti-breast cancer activities of niclosamide, its mechanism of action in adipocyte-breast cancer cell interaction. Cancer-associated adipocytes can create a paracrine loop with tumour cells leading to the induction of EMT, with increased cell migration and invasion^19,46^. Our study provides evidence that human-adipocyte CM can induce EMT in breast cancer cells through the IL-6/STAT3 signalling pathway, perturbation of IL-6/STAT3 signalling with niclosamide inhibit STAT3 phosphorylation and reverse adipocyte-induced EMT in breast cancer cells. Niclosamide also inhibit breast cancer cell motility via a mechanism that involved destabilization of focal adhesion complex formation.

We have recently reported that the ability of human adipocytes to induce EMT in breast cancer cells through the activation of the IL-6/STAT3 signalling, increasing their migratory and invasion potential^19^. Hence, identifying drugs that can inhibit IL-6/STAT3 signalling may present an opportunity to inhibit adipocyte-induced EMT and invasion in breast cancer cells. We found that niclosamide can inhibit human preadipocyte differentiation in a dose-dependent manner and resulted in significant delipidation in differentiated adipocytes (Fig. S1). Adipocytes differentiation is a highly regulated process requiring the activation of several genes and transcription factor such as PPARγ and C/EBPα. The ability of niclosamide to inhibit adipogenesis indicates a potential to block the activation of key gene involved in adipocyte differentiation. This observation is consistent with studies in diabetes and obesity where treatment of mice models with niclosamide induces mitochondrial uncoupling and inhibit adipogenesis^47,48^. However, no studies to this effect has been reported in cancers, thus further studies are required to elucidate the mechanism by which niclosamide inhibit adipogenesis and induced lipolysis in differentiated adipocytes. This may offer an opportunity to use niclosamide to differentially inhibit adipocyte differentiation in the tumour microenvironment and limits its effect on tumour growth.

In both breast cancer cells, we found that human adipocyte CM enhanced proliferation, colony formation, migration and invasion. Subsequent treatment with niclosamide significantly inhibits the proliferation, colony formation, migration and invasion characteristics (Figs 2 and 3). Similar results have been reported for niclosamide in different cancers, Ye *et al*. reported a dose-dependent effects of niclosamide on breast cancer cells (MDA-MB-231, MCF-7, MDA-MB-468) viability and proliferation, although IC_50_ value used to achieve such effects were much higher (MCF-7 – IC_50_ 1.05 µM and MDA-MB-468 – IC_50_ 1.88 µM)^42^. The authors also reported similar effects of niclosamide on apoptosis and colony formation, however these effects were observed in 4TI mouse models^42^. In prostate cancer, niclosamide inhibition of migration is proposed to occur through inhibition of the STAT3-AR axis^35^ and in basal breast cancer the effects of niclosamide is proposed to occur through inhibition of the WNT/β-catenin and STAT3 signalling^49^. We hypothesized perturbation of the IL-6/Stat3 axis with niclosamide may reverse adipocyte-induced proliferation, migration and invasion. We show that niclosamide inhibit EMT-TFs SNAIL and TWIST, thus inhibiting EMT and limiting breast cancer proliferation migration and invasion. We further showed that niclosamide inhibition of EMT may occur via inhibition of Stat3 phosphorylation and nuclear localization similar finding have been reported by Londono-Joshi *et al*. in basal breast cancers where treatment with niclosamide decreased pSTAT3 levels^49^. Treatment with niclosamide showed a time dependent decrease in IL-6, total Stat3 and phosphorylated Stat3 (Fig. 4). Based on these findings we speculate that IL-6/STAT3 signalling pathway is a key pathway by which adipocytes drive breast cancer migration and invasion. Perturbation of the IL-6/STAT3 signalling with low concentration of niclosamide effectively inhibit Stat3 phosphorylation and its nuclear localization in a time-dependent manner. Inhibition of Stat3 activity subsequently results in attenuated activation of EMT transcription factors TWIST and SNAIL, inhibiting the induction of EMT in breast cancer cells **(**Fig. 5k**)**. Resulting in decreased breast cancer cell migration and invasion, two key steps required for cancer cell metastasis.

The process of cell migration requires the formation of focal adhesions, which enhances cell adhesion to the extracellular matrix, and allow the cells to generate the needed forces to drive cell migration^50^. This process is regulated by the synchronous assembly/disassembly of FA structures and proteins. Destabilization of the of FAs reduces cells adhesion to the ECM and limits cell migration^51^. Phosphorylation and activation of FAK, results in FAK phosphorylating several components of the FA complex including paxillin^52^. In both wound healing and transwell migration assay (Figs 3 and 6a) the presence of niclosamide significantly limited cell migration in breast cancer cells, hence we hypothesized that niclosamide inhibition of breast cancer cell migration may involve destabilization of FA complex formation. We observed that human-adipocyte CM increased FA complex formation, with increases in phosphorylated FAK and phosphorylated PAX interaction, treatment with niclosamide significantly impairs FA complex formation after co-culture decreasing pFAK and pPAX levels in a time dependent manner and reducing the interaction between pFAK and pPAX. Our results show that the co-localization ratio of FAK and pPAX at FA complex sites are higher in the presence of adipocyte CM but perturbation with niclosamide destabilises FA complex and deceased FAK and pPAX co-localization. Co-immunoprecipitation results also indicate that niclosamide inhibition of FA complex formation may occur in part via reducing the interaction between key components of the FA complex. Co-cultured breast cancer cells had a higher pFAK and pPAX interaction, however treatment with niclosamide significantly reduced the interaction between pFAK and pPAX. Increased pFAK and pPAX interaction at FA complex sites is linked to FA assembly, disassembly and turnover which allow cellular migration to occur. Increased upregulation of FAK has been reported in breast cancer tissues and identified as playing a critical role breast cancer cell invasion^53^. Various studies have screened for molecules that can impact FA turnover and limit breast cancer cell invasion. In this study we identified niclosamide as a potential inhibitor of FA turnover *in vitro* and linked its activity in part to alteration in FAK and pPAX co-localization, preventing breast cancer cell migration.

In conclusion, this study demonstrates the ability of niclosamide to reverse adipocyte induced EMT in MDA-MB-468 (basal) and MCF-7 (luminal) breast cancer cells in part via inhibition of STAT3 phosphorylation and nuclear localization, reducing breast cancer cell migration and invasion. Our data provide evidence that niclosamide also limit breast cancer cell migration by altering FA turnover. Thus, we offer key insights into the potential of niclosamide as a therapeutic agent in breast cancer microenvironment, although further studies with *in vivo* models are required to determine appropriate concentrations for use in human patients.

## Methods

### Differentiation and collection of human adipocyte conditioned media

Primary human preadipocytes was isolated from white adipose tissue isolated from by-product of human patients with colon cancer and differentiated as described by Lee *et al*.^54^. Briefly, collected adipose tissues were washed with PBS, minced, and digested with type II collagenase (2 mg/ml) in Krebs-Ringer bicarbonate buffer (KRBB) containing 10 mM HEPES (pH 7.4), and 3% BSA for 1 hr at 37 °C. Digested tissues was subsequently passed through a 70 μm cell strainer and centrifuged. Floating adipocytes were collected and pellets containing the stromal vascular (SV) fraction were incubated in red blood cell lysis buffer for 5 min at room temperature. Its then passed through a 40 μm cell strainer, and primary preadipocytes collected by centrifugation at 500 g for 5 min. The resultant cell preparations were subjected to immunostaining or flow cytometry. Primary human preadipocytes are expanded in growth medium Dulbecco’s modified Eagle’s medium (DMEM; Welgene, Inc. Seoul, Korea) with 10% fatal bovine serum (FBS; Gibco TM, Life Technologies, Carlsbad, CA, USA) and 1% penicillin-streptomycin (Gibco TM). Cells were differentiated in adipogenic differentiation medium [DMEM with 5% FBS and 1% Penicillin-streptomycin supplemented with 2.5 mM isobutylmethylxanthine (IBMX) (Sigma-Aldrich, St Louis, MO, USA), 1 μM dexamethasone (Sigma-Aldrich), 1 μg/ml insulin (Sigma-Aldrich)] for 12 days. Cells were subsequently maintained in medium containing insulin for up to 2 weeks. After which cells are rinsed with PBS and replaced with media without differentiation factors and cultured at 37 °C for 48 hours. After 48 hours the media is collected and centrifuged at −4 °C. Supernatant is collected and stored at −20 °C until used. After differentiation, differentiated adipocytes was rinsed with PBS and replaced with media without differentiation factors and cultured at 37 °C for 48 hours. After 48 hours the media is collected and centrifuged at −4 °C. Supernatant is collected and stored at −20 °C until used. The informed consent was acquired from patients and all methods and experimental protocols using human tissue were carried out in accordance with relevant guidelines and regulations approved by the Institutional Review Board of Severance Hospital, Yonsei University Health System (2016-0576-001).

### Niclosamide preparations

Niclosamide (N3510 – Sigma-Aldrich) was dissolved initially as a 10 mM stock solution in dimethyl sulfoxide (DMSO) and for *in-vitro* experiments the stock solution was diluted in serum free media to 20 μM and used for various assays. For vehicle control equal volume of DMSO for 0.250 μM niclosamide was used.

### Oil Red O staining and quantification

Intracellular lipid content of differentiated adipocytes was evaluated by Oil Red O staining. Cells are fixed with 4% paraformaldehyde for 20 minutes at room temperature (RT), rinsed trice with PBS, and stained for 30 minutes with Oil Red O in isopropanol. Images are acquired using the Olympus BX53 microscope (Olympus Optical Co., Tokyo, Japan). For lipid quantification, Oil red O stain is extracted with 100% isopropanol for 5 minutes with gentle rocking. 250 µl of extracted oil red O is transferred into a 96-well plate and measured spectrophotometrically at 492 nm (Tecan Group limited, Männedorf, Switzerland).

### Cell culture of breast cancer cells

The human breast cancer cell lines MDA-MB-468 (Estrogen receptor (ER) negative, Progesterone receptor (PR) negative and Human epidermal growth factor receptor-2 (HER2) negative, basal type) was cultured in DMEM mixed with F12 (DMEM/F12; Welgene) supplemented with 10% FBS and 1% penicillin-streptomycin and MCF-7 (ER, PR positive and HER2 negative, luminal type) was cultured in DMEM/F12 supplemented with 10% FBS, 1% penicillin-streptomycin and 0.1 mg/ml insulin, in a humidified 5% CO_2_ atmosphere. Cultured cells at 70–80% confluence was used in experiments.

### Reverse transcription-quantitative PCR (qtPCR)

Total RNA of cells culture in complete media with/without niclosamide (0.250 µM) and in 75% adipocyte conditioned media with and without niclosamide (0.250 µM) for 48 hours was isolated using the RNeasy Kit (Qiagen, Valencia, CA, USA) following manufacturer’s instruction. Real time PCR was performed with 50 ng of RNA using the One Step SYBR PrimeScript^TM^ RT-PCR kit (Takara Shuzo Co., Japan) according to the manufacturer’s instruction and analysed with the StepOne Plus Real-time PCR system (Applied Biosystems, Foster City, CA, USA). All reactions were performed in triplicate; with the housekeeping gene glyceraldehyde 3-phosphate dehydrogenase (GAPDH) as an internal control mRNA. All primers were initially evaluated for efficiency using the Relative standard curve and the relative gene expression evaluated by comparative CT method (2^−∆∆CT^). Primer sequences are listed in Table 1.

**Table 1:** Primer sequences used for qtPCR.

Primer	Sequence
IL-6	CCAGCTATGAACTCCTTCTC GCTTGTTCCTCACATCTCTC
SNAIL	CACCTCCAGACCCACTCAGAT CCTGAGTGGGGTGGGAGCTTCC
MMP9	CCTGCCAGTTTCCATTCATC GCCATTCACGTCGTCCTTAT
TWIST	CCACGCTGCCCTCGGACAAG CCAGGCCCCCTCCATCCTCC
N-Cadherin	GCGTCTGTAGAGGCTTCTGG GCCACTTGCCACTTTTCCTG
E-Cadherin	CTGAGAACGAGGCTAACG TTCACATCCAGCACATCC
STAT3	TGAGACTTGGGCTTACCATTGGGT TCTTTAATGGGCCACAACAGGGCT
FAK	AATACGGCGATCATACTGGG CATGCCTTGCTTTTCGCTGT
Paxillin	TGGACAGCCCTACTGTGAAA AGAAGTGTTCAGGGTGCCA
GAPDH	ACCCACTCCTCCACCTTTGA CTGTTGCTGTAGCCAAATTCGT

### Co-immunoprecipitation

Co-immunoprecipitation (co-IP) was done using the Thermo Scientific Pierce co-IP kit following the manufacturer’s protocol. Cultured cells were lysed, and total protein harvested using ice-cold non-denaturing lysis buffer (Thermo Scientific, Rockford, IL), 1 mg protein lysate was pre-cleared by incubating with control agarose resin for 1 h at 4 °C. Briefly, 2 μg phosphorylated FAK antibody (Abcam) was immobilized for 2 h using AminoLink Plus coupling resin. The resin was incubated with 500 µg protein lysate overnight at 4 °C. After incubation, the resin was washed, and protein eluted. Samples were analysed by Western blotting using mouse monoclonal anti-paxillin (Abcam Inc., Cambridge, MA), rabbit polyclonal anti-phospho-paxillin (Tyr31) and horseradish peroxidase-conjugated secondary antibodies.

### Western blotting

Cells were lysed in RIPA buffer (150 mM sodium chloride, 1% triton X-100, 1% sodium deoxycholate, 0.1% SDS, 50 mM Tris-HCl, pH 7.5 and 2 mM EDTA) (GenDEPOT, TX, USA) containing 1% protease inhibitor cocktail (GenDEPOT). 20 μg of each sample is separated by SDS-PAGE and transferred to a nitrocellulose membrane (GE Healthcare, Chalfont St Giles, UK). Western blotting was performed as previously described by Liu *et al*.^55^.

### Immunofluorescence staining

Cells were seeded on cover slide coated with poly-L-lysine and cultured with/ without adipocyte conditioned media for 48 hr. Cells was rinsed in PBS and fixed with 4% paraformaldehyde, permeabilized with 0.2% Triton X-100 and stained with appropriate primary antibodies. For double staining experiments, antibodies were diluted together and incubated with cells overnight at 4 °C. Goat Anti-Rabbit IgG (Alexa Fluor 647) and Goat Anti-mouse IgG (Alexa Fluor 488) antibodies (Abcam) were used as secondary antibodies. Counter staining of cell nuclei was performed using DAPI (Invitrogen, Carlsbad, CA, USA). Stained cells were visualized using the ZEISS LSM 710 microscope (ZEISS, Germany).

### Generation of STAT3 reporter cells

MDA-MB-468 and MCF-7 were transfected with STAT3 reporter plasmid (Cignal Lenti STAT3 Reporter, QIAGEN, Hilden, Germany) using SureENTRY transduction reagent (Qiagen). Stable STAT3 reporting MDA-MB-468 and MCF-7 cells were selected with 10 µg/ml and 8 µg/ml of puromycin (Sigma) respectively over 10 days to generate stable STAT3 reporter MDA-468 and MCF-7 cell lines.

### STAT3 Luciferase reporter assays

Stable STAT3 reporter MDA-MB-468 and MCF-7 breast cancer cells were seeded at 1 × 10^6^ cells in a 100 mm and cultured for 12 hrs and treated with 0.250 µM niclosamide is added and incubated at 37 °C, STAT3 promoter activity is measured at specific time points (0, 1, 6, 12, 24 and 48 hours). STAT3 promoter activity was determined by Promega Dual-Luciferase reporter assay system (Promega corporation, Madison, USA) following manufacturers instruction and luciferase activity measured in the Tecan™ microplate-Luminometer (Tecan, Switzerland). The constitutively expressed non-inducible Renilla luciferase activity served as internal control for normalizing transfection efficiencies.

### Statistical analysis

Data were analysed, and graphs plotted with GraphPad Prism version 6 software (GraphPad Inc.). Student’s t-test was used to compare differences between two groups and multiple analysis was performed using analysis of variance (ANOVA). Multiple analysis of groups was checked for after ANOVA using Bonferroni’s multiple comparison test. Statistical significance was defined as P < 0.05. For co-localization experiments, using ImageJ Pearson correlation coefficients were calculated. The Pearson’s correlation coefficient reflects the degree of linear relationship between two fluorescence intensities. Co-localization coefficient >0.5 was defined as positive correlation, and 0.5 or less as no co-localization.

## Supplementary information

Supplement information
