# Supplementary material for: Niclosamide reverses adipocyte induced epithelial-mesenchymal transition in breast cancer cells via suppression of the interleukin-6/STAT3 signalling axis
Source: Sci Rep. 2019 Aug 5;9:11336. doi: 10.1038/s41598-019-47707-2 (PMC6683291; doi:10.1038/s41598-019-47707-2)
Supplement: Supplementary file 1 — Supplement information [file 41598_2019_47707_MOESM1_ESM.pdf]

Supplementary Information

Niclosamide reverses adipocyte induced epithelial-mesenchymal transition in breast cancer cells via suppression of the interleukin-6/STAT3 signalling axis.

[1]Jones Gyamfi, [1] Yun-Hee Lee, [2] Byung Soh Min, [1]Junjeong Choi*

[1] College of Pharmacy, Yonsei Institute of Pharmaceutical Sciences, Yonsei University, Incheon, Korea

[2] Department of Surgery, Yonsei University College of Medicine, 50 Yonsei-ro, Seodaemun-gu, Seoul 120-752, Republic of Korea

Corresponding author:

Junjeong Choi M.D., Ph. D.

College of Pharmacy, Yonsei Institute of Pharmaceutical Sciences, Yonsei University, Incheon, Korea

Veritas Hall D 306, 85 Songdogwahak-ro,
Incheon, 21983, Republic of Korea

Phone: +82-32-749-4521

Fax: +82-32-749-4105

E-mail: junjeong@yonsei.ac.kr

SUPPLEMENTARY FIGURE 1

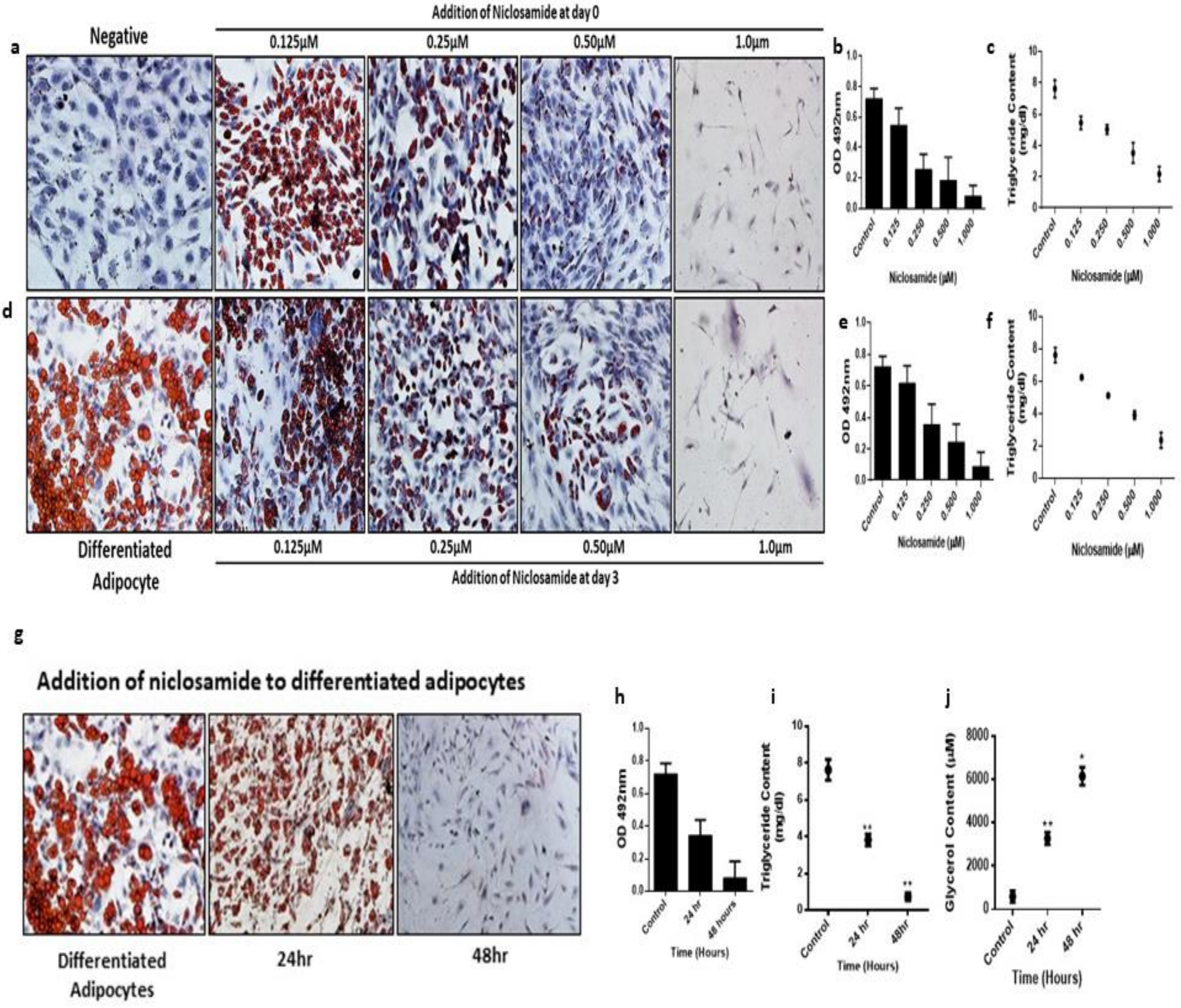

Supplementary figure 1: Niclosamide inhibits human preadipocytes differentiation. (a-c) Evaluation of the differentiation status in human preadipocytes treated with niclosamide from day 0, **a**. Representative images of Oil Red O staining shown at x200 magnification. **b.** Quantitative analysis of Oil Red O staining. **c.** Quantitative analysis of triglyceride content. **(d-f)** Evaluation of the differentiation status in human preadipocytes treated with niclosamide from day 3 after induction of differentiation, **d.** Representative images of Oil Red O staining shown at x200 magnification, **e.** Quantitative analysis of Oil Red O staining and **f.** Quantitative analysis of triglyceride content. **(g-j)** Evaluation of the effects of niclosamide in differentiated human adipocytes: **g.** Representative images of Oil Red O staining shown at x200 magnification **h.** Quantitative analysis of Oil Red O staining **i.** Quantitative analysis of triglyceride content and **j.** quantitative analysis of glycerol levels.
(Data indicate mean ± SD; ***p< 0.001; **p<0.01; *p<0.05).

SUPPLEMENTARY FIGURE 2

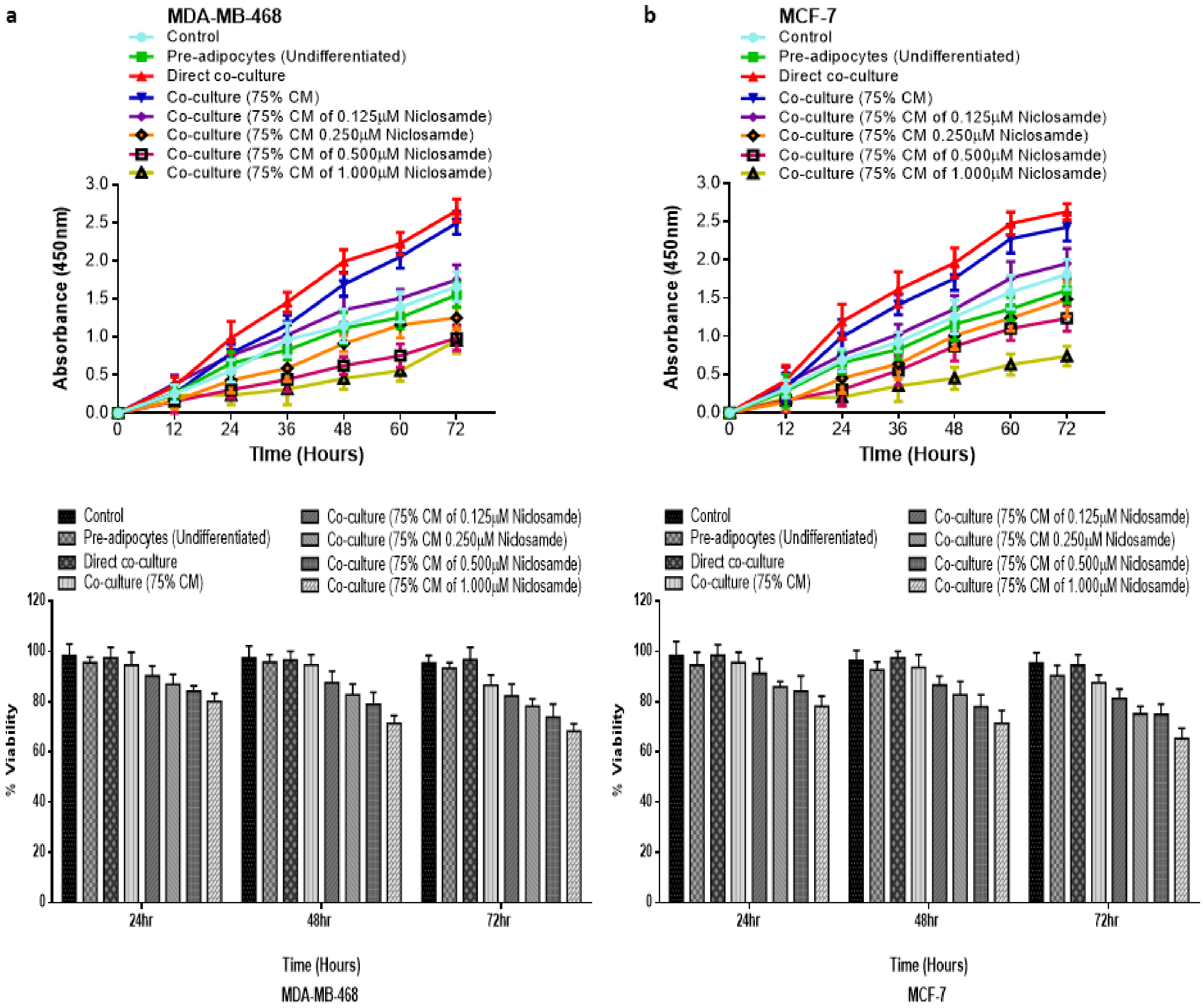

Supplementary Figure 2: Effect of different adipocyte differentiation status on breast cancer cells. (a, b) Quantitative analysis on the effect of adipocyte differentiation status on breast cancer cells proliferation. Different concentrations of niclosamide (0.000, 0.125, 0.250, 0.500 and 1,000) was added to human preadipocyte 3 days after differentiation induction. 75% adipocyte CM was added to MDA-MB-468 and MCF-7 breast cancer cells and proliferation rate assayed at different time intervals (0, 12, 24, 36, 48, 60 and 72 hours). **(c, d)** Quantitative analysis on the effect of adipocyte differentiation status on breast cancer cells viability. Different concentrations of niclosamide (0.000, 0.125, 0.250, 0.500 and 1,000) was added to human preadipocyte 3 days after differentiation induction. 75% adipocyte CM was added to MDA-MB-468 and MCF-7 breast cancer cells and viability of cancer cells assayed at different time intervals (24hr, 48hr and 72 hours).

SUPPLEMENTARY FIGURE 3

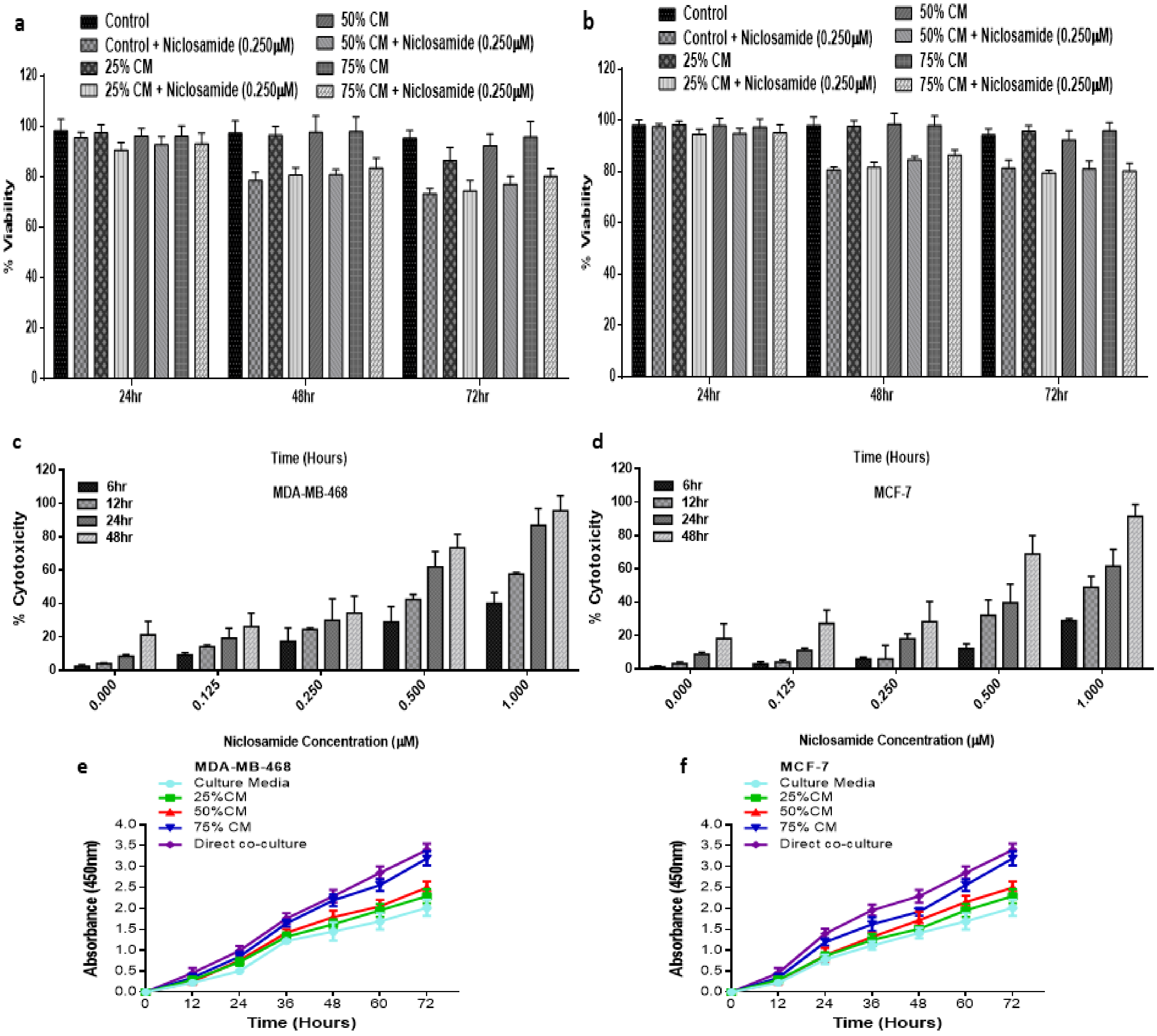

Supplementary Figure 3: Effect of adipocyte conditioned media on breast cancer cells. **(a, b)** Quantitative analysis of the effects of different concentrations (25%, 50%, 75%) of human adipocyte conditioned media (CM) on MDA-MB-468 and MCF-7 breast cancer cell viability at different time intervals (24, 48 and 72 hours) with/ without niclosamide (0.250µM). **(c, d)** Quantitative analysis of the cytotoxicity of different concentrations of niclosamide (0.000, 0.125, 0.250, 0.500 and 1,000) on MDA-MB-468 and MCF-7 breast cancer cells at different time intervals (24, 48 and 72 hours). **(e, f)** Quantitative analysis on the effect of different concentration of human adipocyte CM on MDA-MB-468 and MCF-7 breast cancer cells proliferation at different time intervals.

SUPPLEMENTARY FIGURE 4

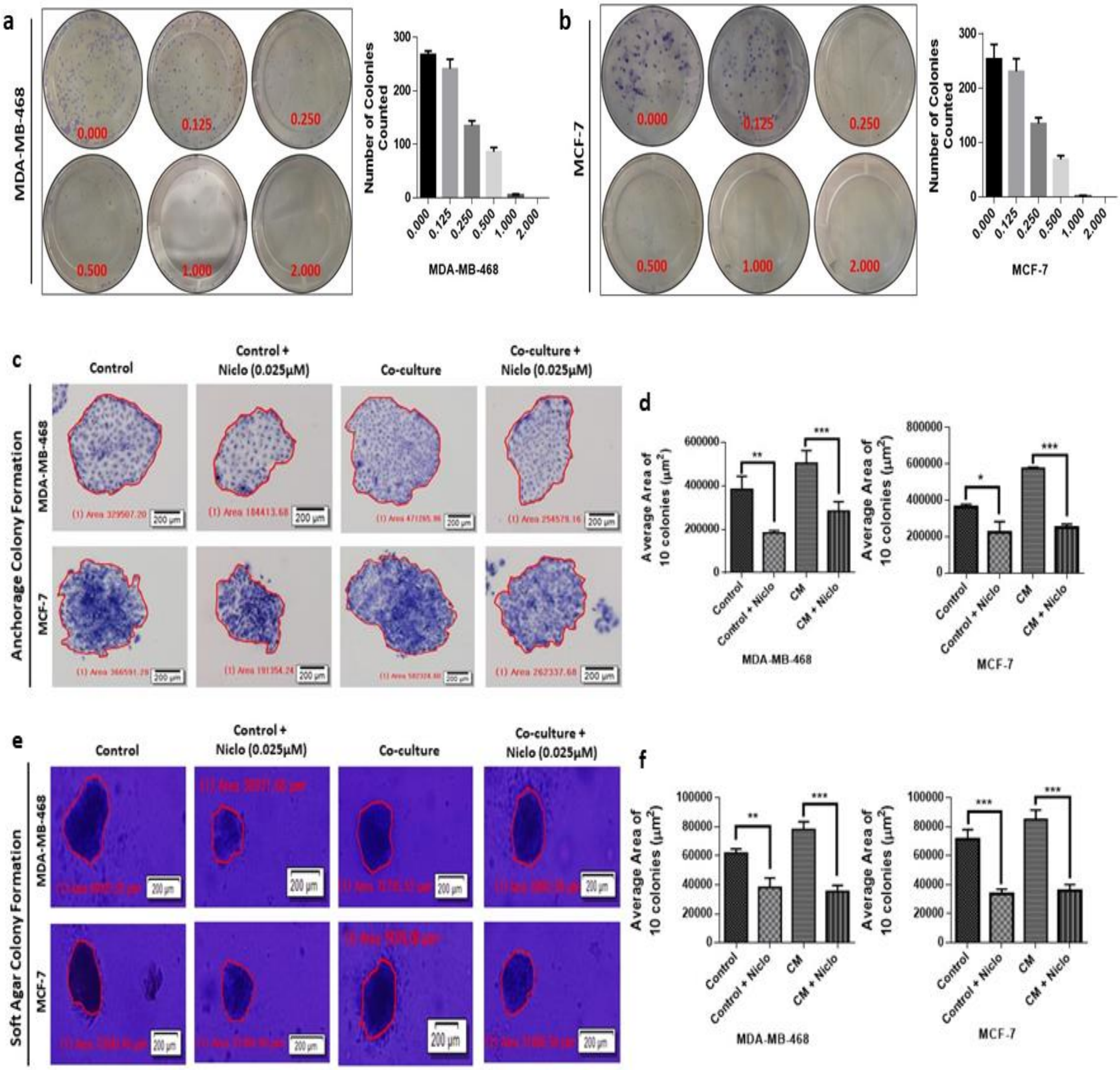

Supplementary Figure 4: Effect of niclosamide on breast cancer cells colony formation. (a, b) Image and quantitative analysis of the effects of different concentrations of niclosamide (0.000, 0.125, 0.250, 0.500, 1.000 and 2.000μM) on MDA-MB-468 and MCF-7 breast cancer cell ability to form colonies after culturing for 14 days **(c, d, e and f)** Image and quantitative analysis of the effects of 0.250μM niclosamide on the size of colonies formed by MDA-MB-468 and MCF-7 breast cancer cells with **(e)**/without **(c)** soft agar. Average area size of 10 colonies were estimated by using the CellSens software (Olympus). Colonies formed in plates without soft agar were generally larger than with soft agar. Niclosamide significantly decreased the size of colonies formed with/without adipocyte conditioned medium. (Data indicate mean ± SD; ***$p < 0.001$; **$p<0.01$; *$p<0.05$).

SUPPLEMENTARY FIGURE 5

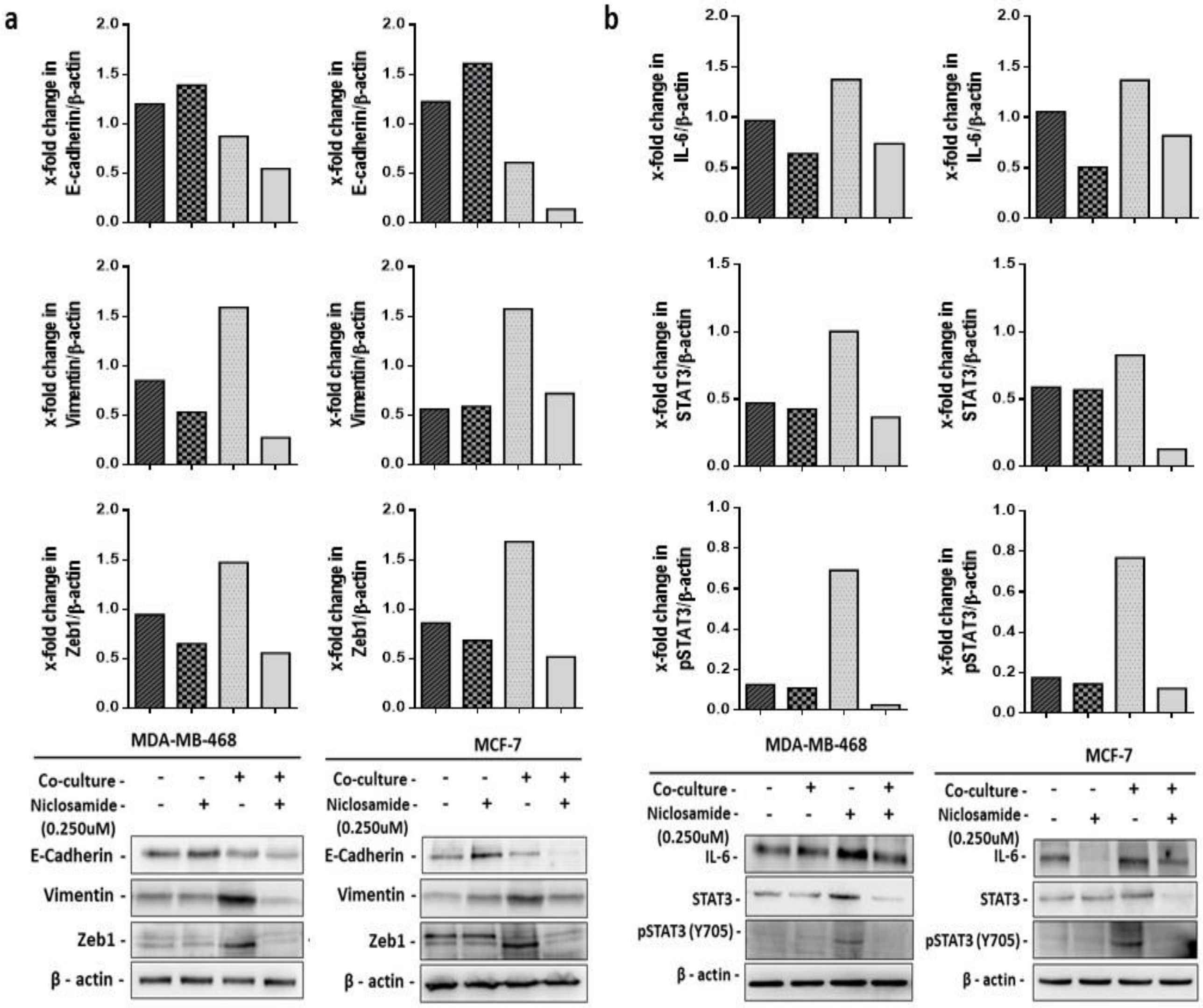

Supplementary Figure 5: (a) Niclosamide reverses adipocyte-induced EMT-phenotype. Representative western blot images and quantification showing levels of E-cadherin, Zeb1 and vimentin in MDA-MB-468 and MCF-7 breast cancer cells cultured with/without adipocyte-CM and with/without 0.250µM niclosamide. **(b) Niclosamide inhibits adipocyte-induced activation of IL-6/STAT3 signalling axis.** Representative western blot images and quantification showing levels of IL-6, STAT3 and phosphorylated STAT3 (Y705) in MDA-MB-468 and MCF-7 breast cancer cells cultured with/without CM and with/without 0.025µM niclosamide.

SUPPLEMENTARY FIGURE 6

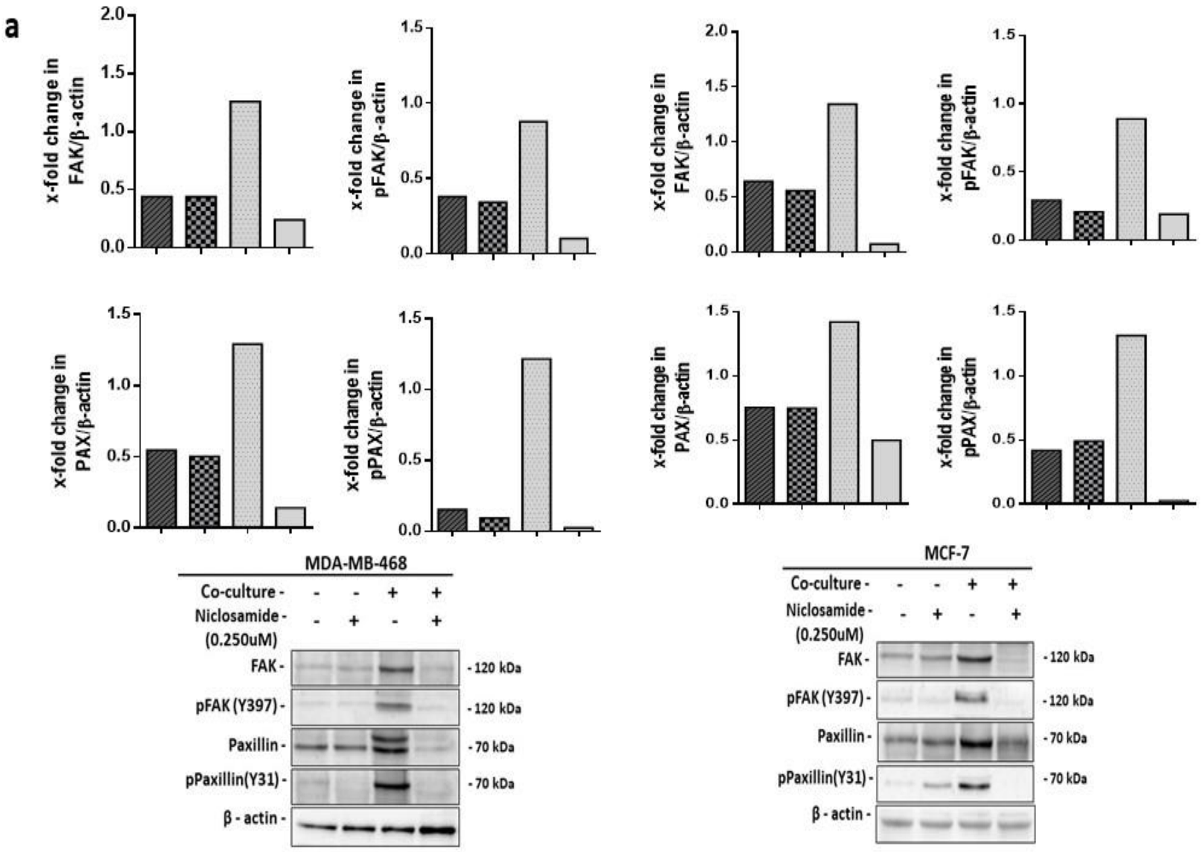

Supplementary Figure 6: (a) Niclosamide destabilizes focal adhesion complex formation. Representative western blot images and quantification showing levels of FAK, phosphorylated FAK(Y397), paxillin and phosphorylated paxillin (Y31) in MDA-MB-468 and MCF-7 breast cancer cells cultured with/without CM and with/without 0.250µM niclosamide.

SUPPLEMENTARY FIGURE 7

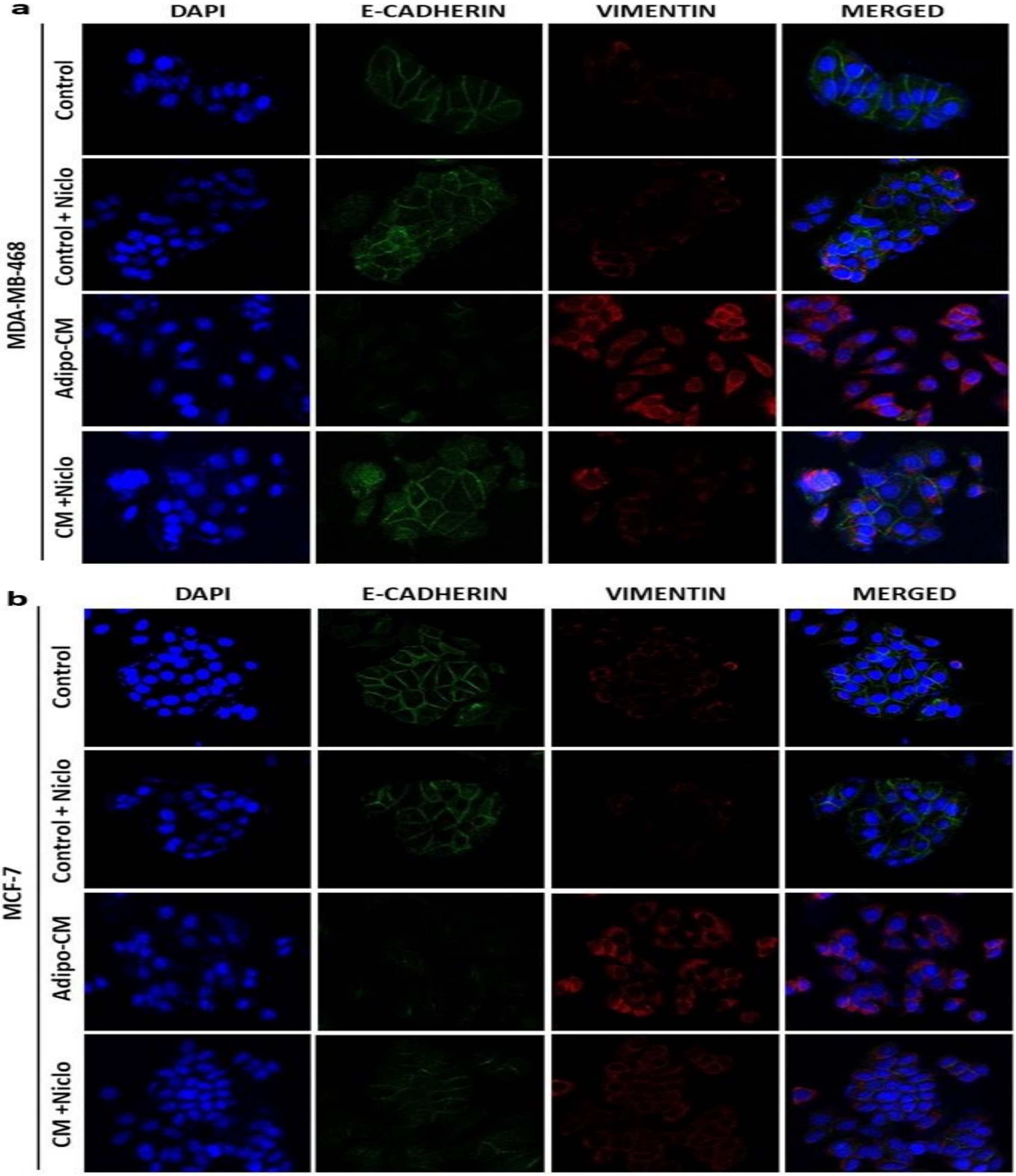

Supplementary Figure 7: Representative confocal images of immunofluorescent staining of E-cadherin (green) and Vimentin (red) co-stained with nuclear marker DAPI (blue) in breast cancer cells cultured with/without adipocyte CM and with/without niclosamide.

SUPPLEMENTARY FIGURE 8

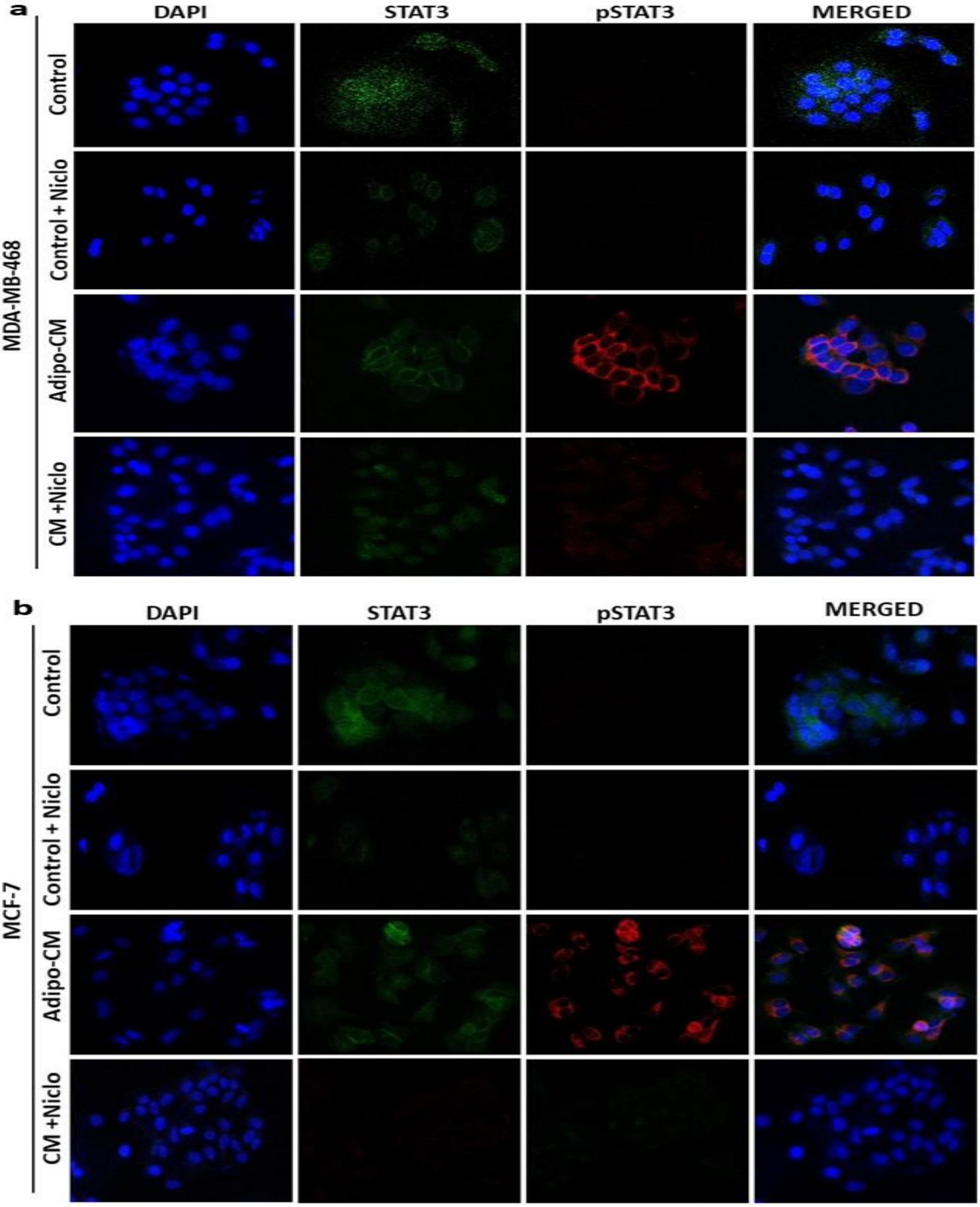

Supplementary Figure 8: Representative confocal images of immunofluorescent staining of STAT3 (green) and phosphorylated STAT3(Y705) co-stained with nuclear marker DAPI (blue) in breast cancer cells cultured with/without adipocyte CM and with/without niclosamide.

SUPPLEMENTARY FIGURE 9

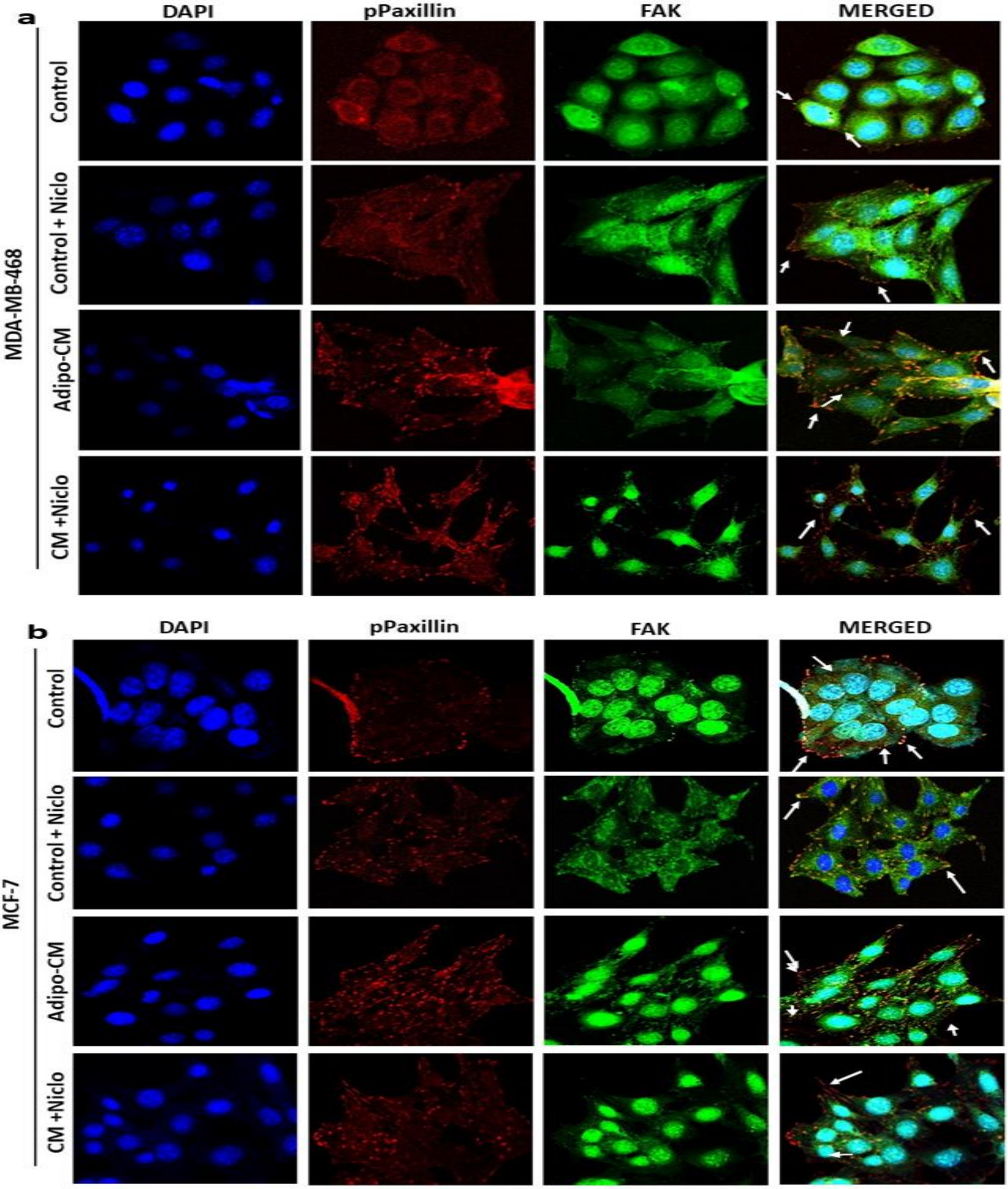

Supplementary Figure 9: Representative confocal images of immunofluorescent staining of phosphorylated paxillin (red) and FAK (green) co-stained with nuclear marker DAPI (blue) in breast cancer cells cultured with/without adipocyte CM and with/without niclosamide.

SUPPLEMENTARY FIGURE 10

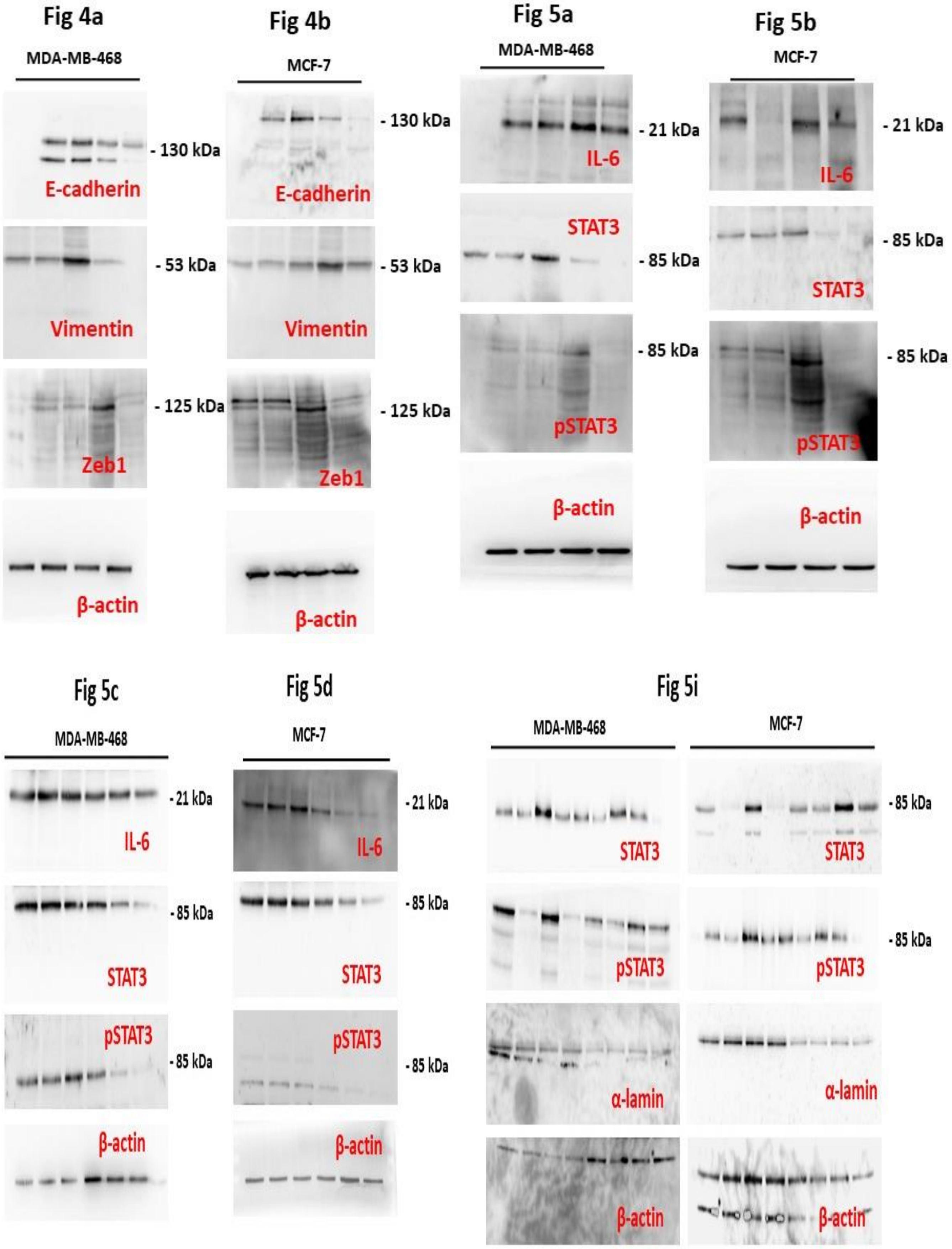

Fig 6c

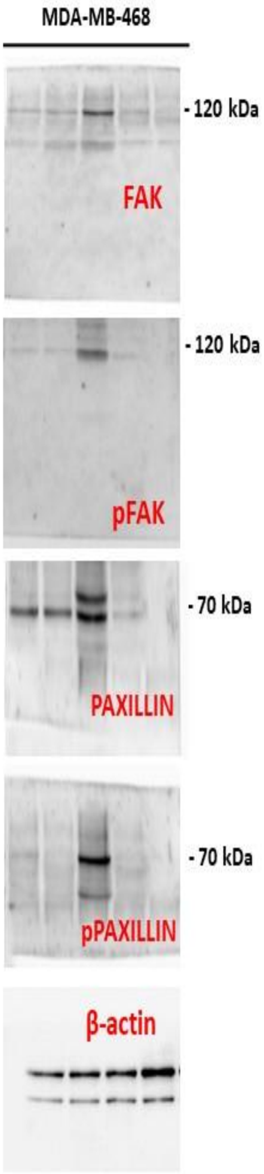

Fig 6d

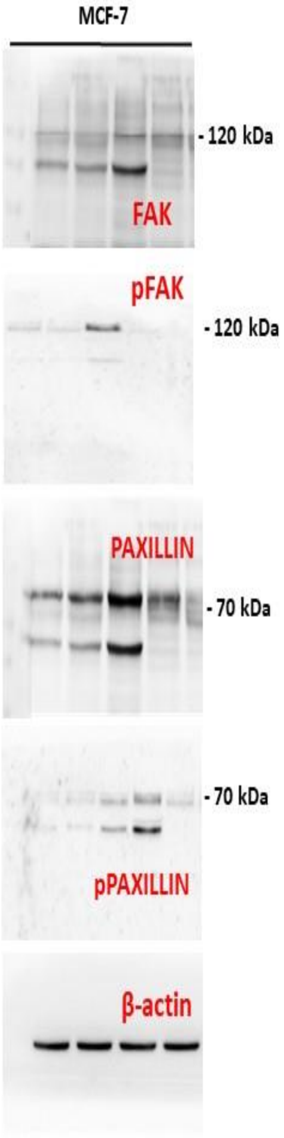

Fig 6g

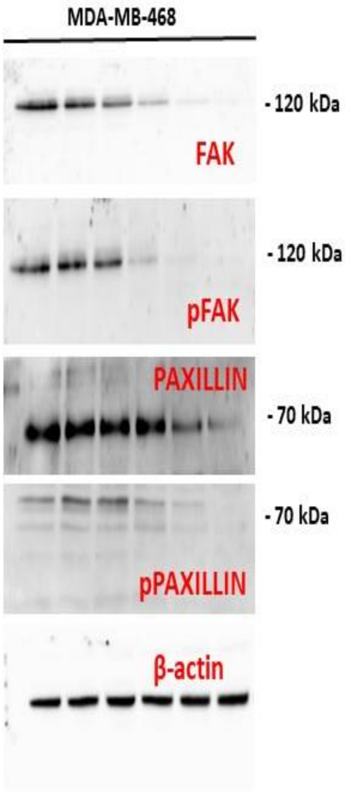

Fig 6h

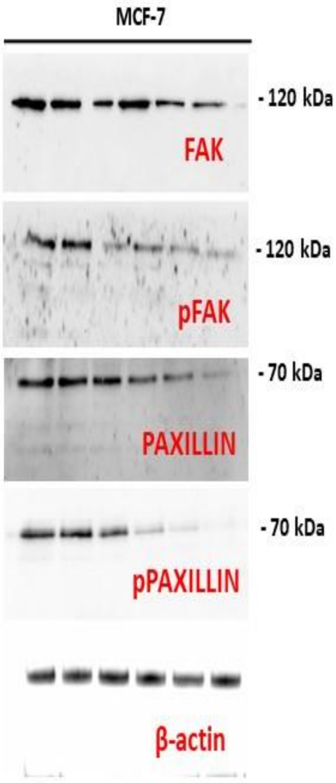

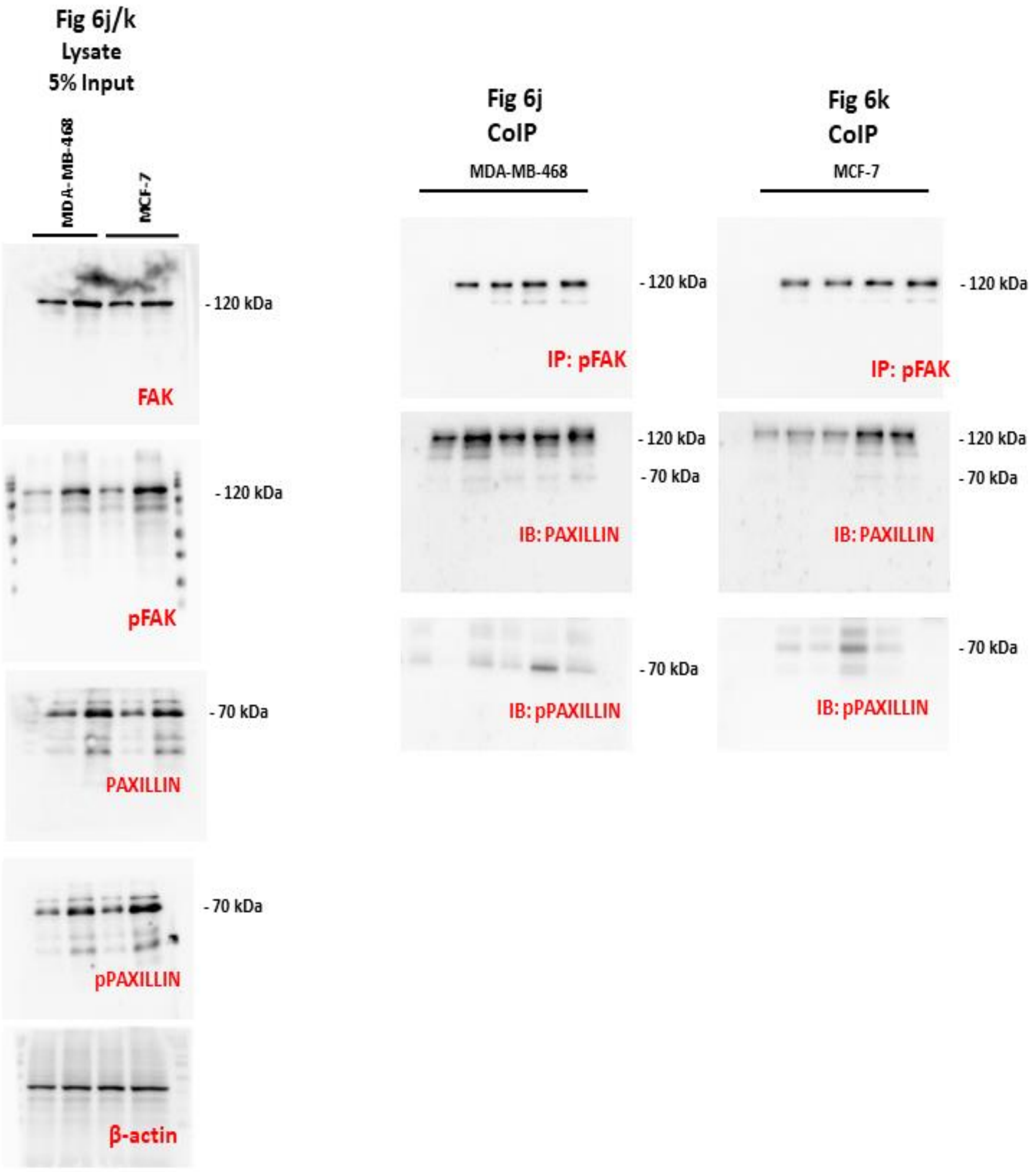

Supplementary Figure 10: Uncropped scans of western blot displayed in Fig. 4a, 4b, 5a, 5b, 5c, 5d, 5i, 6c, 6d, 6g, 6h, 6j and 6k.

Extended Experimental Procedures

Differentiation and Collection of human adipocyte conditioned Media

Primary human preadipocytes from white adipose tissue isolated from by-product of human patients with colon cancer as described described by Lee *et al* [1]. Briefly, collected adipose tissues were washed with PBS, minced, and digested with type II collagenase (2 mg/ml) in Krebs-Ringer bicarbonate buffer (KRBB) containing 10 mM HEPES (pH 7.4), and 3% BSA for 1 hr at 37 °C. Digested tissues was subsequently passed through a 70 μm cell strainer and centrifuged. Floating adipocytes were collected and pellets containing the stromal vascular (SV) fraction were incubated in red blood cell lysis buffer for 5 min at room temperature. Its then passed through a 40 μm cell strainer, and primary preadipocytes collected by centrifugation at 500 g for 5 min. The resultant cell preparations were subjected to immunostaining or flow cytometry. Primary human preadipocytes are expanded in growth medium Dulbecco's modified Eagle's medium (DMEM; Welgene, Inc. Seoul, Korea) with 10% fatal bovine serum (FBS; Gibco™, Life Technologies, Carlsbad, CA, USA) and 1% penicillin-streptomycin (Gibco™). Cells were differentiated in adipogenic differentiation medium [DMEM with 5% FBS and 1% Penicillin-streptomycin supplemented with 2.5mM isobutylmethylxanthine (IBMX) (Sigma-Aldrich, St Louis, MO, USA), 1μM dexamethasone (Sigma-Aldrich), 1μg/ml insulin (Sigma-Aldrich)] for 12 days. Cells were subsequently maintained in medium containing insulin for up to 2 weeks. After which cells are rinsed with PBS and replaced with media without differentiation factors and cultured at 37°C for 48 hours. After 48 hours the media is collected and centrifuged at -4°C. Supernatant is collected and stored at -20°C until used. The informed consent was acquired from patients and all methods and experimental protocols using human tissue were carried out in accordance with relevant guidelines and regulations approved by the Institutional Review Board of Severance Hospital, Yonsei University Health System (4-2014-0054).

Niclosamide Preparations

Niclosamide (N3510 – Sigma-Aldrich) was dissolved initially as a 10mM stock solution in dimethyl sulfoxide (DMSO) and for in-vitro experiments the stock solution was diluted in serum free media to 20µM and used for various assays. For vehicle control equal volume of DMSO for 0.250µM niclosamide was used.

Oil Red O Staining and Quantification

Intracellular lipid content of differentiated human adipocytes was evaluated with Oil Red O staining. Differentiated cells are fixed with 4% paraformaldehyde for 20 minutes at room temperature (RT), rinsed trice with water, and stained for 30 minutes with Oil Red O in isopropanol. Cells are rinsed with water and images acquired using the Olympus BX53 microscope (Olympus Optical Co., Tokyo, Japan). For lipid quantification, stained cells are rinsed with water and 60% isopropanol. Oil red O stain is extracted with 100% isopropanol for 5 minutes with gentle rocking. 250µl of extracted oil red O is transferred into a 96-well plate and measured spectrophotometrically at 492nm (Tecan Group limited, Männedorf, Switzerland).

Triglyceride and Glycerol content analysis.

TG content of differentiated adipocytes was quantified using the Cayman triglyceride colorimetric assay kit (Cayman chemical, Ann Arbor, USA) following manufacturers instruction. Results were read spectrophotometrically at 550nm (Tecan Group limited, Männedorf, Switzerland). Measure of lipolysis: Glycerol released by human adipocytes differentiated in different concentration of niclosamide was assessed using the Glycerol-Free Reagent Kit (Sigma-Aldrich) following manufacturer's instructions.

Cell culture of breast cancer cells

The human breast cancer cell lines MDA-MB-468 (Estrogen receptor (ER) negative, Progesterone receptor (PR) negative and Human epidermal growth factor receptor-2 (HER2) negative, basal type) was cultured in DMEM mixed with F12 (DMEM/F12; Welgene) supplemented with 10% FBS and 1% penicillin-streptomycin and MCF-7 (ER, PR positive and HER2 negative, luminal type) was cultured in DMEM/F12 supplemented with 10% FBS, 1% penicillin-streptomycin and 0.1mg/ml insulin, in a humidified 5% CO_2 atmosphere. Cultured cells at 70-80% confluence was used in experiments.

Co-culture, cell migration and invasion assay

A total of 5 x 10^5 breast cancer cells was seeded in a 100mm cell culture dish and cultured at 37^oC. After 12 hours, complete culture media is replaced with 75% human adipocyte conditioned media (CM) with or without niclosamide (0.250µM) (Sigma-Aldrich) diluted in DMSO and cultured for 48 hours. The effect of niclosamide on breast cancer cell migration were evaluated using corning transwell insert (8-µm pore size; Corning Inc.) and invasion with

matrigel-coated transwell insert (1.0 mg/ml matrigel; BD Biosciences, Bedford, MA, USA). Breast cancer cells maintain in complete media with and without niclosamide and in 75% adipocyte CM with and without niclosamide for 48 hours are harvested and seeded at 5 x 10^4 cells in 300µl of serum free media added to the upper chamber with 10% FBS media in the bottom chamber. Cells were cultured in humidified 5% CO_2 atmosphere for an appropriate time (24 hours for migration and 48 hours for invasion), non-invading cells were removed with a cotton swap and invading cells were fixed and stained by the Diff-Quik kit (Sysmex, Seoul, Korea). The number of invading and migrating cells was counted under a microscope in five random fields of each membrane at x100 magnification.

Cell proliferation assay

To determine the cell proliferation rate, breast cancer cell is seeded at 0.25 x 10^4 cells into a 96 well plate and cultured for 12 hours. After 12 hours, media is replaced with complete media with/ without niclosamide (0.250µM) and 75% adipocyte conditioned media with/ without niclosamide (0.250µM) and maintained for the appropriate length of time (24, 48, 72 and 96 hours, all reactions are performed in triplicate. After each time period, 10µl of CCK-8 solution (Dojindo, Kumamoto, Japan) was added to each well and incubated for 2 hours and optical density was measured spectrophotometrically at 450nm (Tecan Group limited, Männedorf, Switzerland).

Cell viability assay

To determine cell viability after niclosamide treatment, breast cancer cell was cultured in complete cell culture media with and without niclosamide (0.250µM) and in 75% adipocytes conditioned media with and without niclosamide (0.250µM) for 48 hours. Cells are seeded at 0.25 x 10^4 in 96 well plate, and cultured for 24 hours, after which niclosamide is added and maintained for 48 hours. Cell viabilities was evaluated using EZ-CYTOX cell viability kit (Douzen, Seoul, Korea), briefly, 10µl of EZ-cytox reagent (DoGEN, Kumamoto, Korea) was added to each well and incubated for 2 hours at 37°C and absorbance measured at 450nm spectrophotometrically (Tecan Group limited, Männedorf, Switzerland).

Cytotoxicity Assay (Lactate Dehydrogenase Assay)

To determine the cytotoxic effect of niclosamide on breast cells, we evaluated the effects of niclosamide at different concentrations (0,000, 0.125, 0.250, 0.400, 0.500 and 1.000 µM) and at different time points (6, 12, 24 and 48 hours) on MDA-MB-468 and MCF-7 breast cancer

cells using the Pierce LDH Cytotoxicity Assay Kit (Thermo Scientific) following manufacturers information. Briefly, cells are seeded in a triplicate in a 96-well plate at 5.0 x10^3 cell/100ul and incubated overnight at 37°C in a 5% CO_2. Growth media is replaced the following day with the different concentrations of niclosamide in 100ul of media to be tested. Triplicate cell wells are included for spontaneous LDH activity controls (water treatment) and Maximal LDH Activity controls (10X lysis Buffer, supplied with kit). The plate was incubated for the different time points at 37°C in a 5% CO_2 incubator. After each indicated time 50µl of each sample medium and control medium are transferred to a new 96-well plate in triplicate. To each well 50µl of reaction mixture is added and mixed by gentle tapping and incubated at room temperature for 30 minutes protected from light. After, 50µl of stop solution is added to each well and mixed gently. Absorbance was measured at 490nm and 680nm, LDH activity was determined by subtracting absorbance at 680nm from absorbance at 490nm and percentage of cytotoxicity was calculated using manufacturers equation.

Wound-healing assay

MDA-MB-468 and MCF-7 cells are seeded at 5 x 10^5 into 6-well plates and cultured in complete cell media into a monolayer. At 90% confluence, complete media is replaced with serum free media and cells are cultured overnight until a monolayer is formed. Using a sterile 100µl pipette tip a linear scratch was made on the cell monolayer. Cells are washed with cell culture media and cultured with complete media with/ without niclosamide and 75% adipocyte conditioned media with/ without niclosamide (0.250µM). Photomicrograph was taken of migrated cells at 0 and 48 hours at x100 magnification. The captured images were analysed using the TScratch software. All experiments were repeated at least three times.

Annexin V-FITC Apoptosis Assay

MDA-MB-468 and MCF-7 cells are seeded at 4 x 10^5 cells per well and cultured for 24 hours at 37°C after which cells are treated with different concentrations of niclosamide (0, 0.125, 0.250, 0.500 and 1.000 µM) for 24 hours. Cells are then stained using Alexa Fluor 488 annexin V/ Dead cell apoptotic kit (Invitrogen, Paisley, UK) following manufacturers instruction. Apoptotic cells were detected by flow cytometry using BD FACSAria™ III (BD Biosciences, San Jose, USA).

Clonogenic Assays

MDA-MB-468 and MCF-7 cell were seeded at 1000 cells per ml in 6-well plates and incubated at 37°C in culture media with/ without niclosamide (0.250µM) and in 75% adipocyte conditioned media with/ without niclosamide (0.250µM) for 14 days [2]. Cell culture media was changed every 3 days. After day 14, cells are rinsed with PBS, fixed with acetic acid and methanol (1:3), and stained with 0.05% crystal violet.

To determine the effect of niclosamide on MDA-MB-468 and MCF-7 clonogenicity, cells are 1000 cells per ml in 6-well plates and incubated at 37°C in culture media with different concentrations of niclosamide (0.000, 0.125, 0.250, 0,500, 1.000 and 2.000µM) for 14 days. Cell culture media was changed every 3 days. After day 14, cells are rinsed with PBS, fixed with acetic acid and methanol (1:3), and stained with 0.05% crystal violet. Images are captured, and number of colonies formed counted using ImageJ software. Size of colonies was determined with the CellSens software (Olympus Optical Co.) by estimating the average area of 10 colonies at 40X magnification.

Soft Agar Assay

Soft agar colony forming assay was performed using 2,500 cells for each plate, as described previously [3]. Briefly, 48 hr after culturing cells with/ without niclosamide (0.250µM) and in 75% adipocyte conditioned media with/ without niclosamide (0.250µM) cells were mixed with DMEM containing soft agar (Difco, Mubai, India) to a final concentration of 0.35%. These cells were then plated in a 6 well plate coated with 0.6% soft agar in DMEM. After 14 days, the number of colonies in three random microscopic fields were counted, and the images were captured using an Olympus BX53 microscope (Olympus Optical Co., Tokyo, Japan). Size of colonies was determined with the CellSens software (Olympus Optical Co.) by estimating the average area of 10 colonies at 40X magnification.

Reverse transcription-quantitative PCR (qtPCR)

Total RNA of cells culture in complete media with and without niclosamide (0.250µM) and in 75% adipocyte conditioned media with and without niclosamide (0.250µM) for 48 hours was isolated using the RNeasy Kit (Qiagen, Valencia, CA, USA) following manufacturer's instruction. Real time PCR was performed with 50ng of RNA using the One Step SYBR PrimeScript^{TM} RT-PCR kit (Takara Shuzo Co., Japan) according to the manufacturer's instruction and analysed with the StepOne Plus Real-time PCR system (Applied Biosystems, Foster City, CA, USA). All reactions were performed in triplicate; with the housekeeping gene

glyceraldehyde 3-phosphate dehydrogenase (GAPDH) as an internal control mRNA. All primers were initially evaluated for efficiency using the Relative standard curve and the relative gene expression evaluated by comparative CT method ($2^{-\Delta\Delta CT}$). Primer sequences are listed in

Co-immunoprecipitation

Co-immunoprecipitation (co-IP) was done using the Thermo Scientific Pierce co-IP kit (Pierce Biotechnology, Rockford, USA) following the manufacturer's protocol. Cultured cells were lysed, and total protein harvested using ice-cold non-denaturing lysis buffer (Thermo Scientific, Rockford, IL), 1mg protein lysate was pre-cleared by incubating with control agarose resin for 1h at 4°C. Briefly, 2μg phosphorylated FAK antibody (Abcam) was immobilized for 2h using AminoLink Plus coupling resin. The resin was incubated with 500μg protein lysate overnight at 4°C. After incubation, the resin was washed, and protein eluted. Samples were analysed by Western blotting using mouse monoclonal anti-paxillin (Abcam Inc., Cambridge, MA), rabbit polyclonal anti-phospho-paxillin (Tyr31) and horseradish peroxidase-conjugated secondary antibodies.

Western blotting

Cells were lysed in RIPA buffer (150mM sodium chloride, 1% triton X-100, 1% sodium deoxycholate, 0.1% SDS, 50mM Tris-HCl, pH 7.5 and 2mM EDTA) (GenDEPOT, TX, USA) containing 1% protease inhibitor cocktail (GenDEPOT). 20μg of each sample is separated by SDS-PAGE and transferred to a nitrocellulose membrane (GE Healthcare, Chalfont St Giles, UK). Western blotting was performed as previously described by Liu *et al* [4]. with primary antibodies to mouse anti-E-cadherin 1:1000 (Cell Signalling, Danvers, MA, USA), rabbit anti-Vimentin 1:500 (Abcam, Cambridge, UK), rabbit anti-Zeb1 1:500 (Sigma), rabbit anti-IL-6 1:1000 (Abcam), rabbit anti-STAT3 1:500 (phosphor Y705: Abcam), mouse anti-STAT3 1:1000 (Cell signalling), mouse anti-FAK 1:500 (Santa Cruz Biotechnology, Santa Cruz, USA), rabbit anti-FAK 1:500 (phospho Y397, abcam), mouse anti-Paxillin 1:1000 (ThermoFisher, Rockford, USA), rabbit anti-Paxillin 1:500 (phosphor Tyr31, ThermoFisher), rabbit anti-α-lamin 1:1000 and goat anti-β-actin 1:5000 (Santa Cruz Biotechnology, Santa Cruz, USA). All primary antibodies were diluted in 5% Bovine Serum Albumin (BSA) in Tris-buffered saline (TBS) containing 0.1% Tween-20 (TBST) and incubated overnight at 4^{0}C. Secondary antibody included goat anti-rabbit IgG-HPR 1:5000 (Santa Cruz Biotechnology), goat anti-mouse IgG-HPR 1:5000 (Santa Cruz Biotechnology) and rabbit anti-goat IgG-HPR 1:5000 (GenDepot,

TX, USA). Protein bands were visualised using enhanced chemiluminescence reagents (Western Lighting Plus, PerkinElmer, USA).

Immunofluorescence staining

Cells were seeded on cover slides coated with poly-L-Lysine cultured with/ without adipocyte conditioned media in the presence/absence of niclosamide for 48hr. Cells was rinsed in PBS and fixed with 4% paraformaldehyde, permeabilized with 0.2% Triton X-100 and stained with appropriate primary antibodies. For double staining experiments, antibodies were diluted together and incubated with cells overnight at 4°C. Goat Anti-Rabbit IgG (Alexa Fluor 647) and Goat Anti-mouse IgG (Alexa Fluor 488) antibodies (Abcam) were used as secondary antibodies. Counter staining of cell nuclei was performed using DAPI (Invitrogen, Carlsbad, CA, USA). Stained cells were visualized using the ZEISS LSM 710 microscope (ZEISS, Germany).

Antibodies used included mouse anti-E-cadherin 1:500 (Cell Signalling, Danvers, MA, USA), rabbit anti-Vimentin 1:500 (Abcam, Cambridge, UK), mouse anti-STAT3 (dilution 1:200) (Cell Signalling Technology), rabbit anti-P-STAT3(Y705) (dilution 1:200) (Abcam), mouse anti-FAK 1:200 (Santa Cruz Biotechnology, Santa Cruz, USA), rabbit anti-FAK 1:200 (phospho Y397, abcam), mouse anti-Paxillin 1:500 (ThermoFisher, Rockford, USA), and rabbit anti-Paxillin 1:500 (phosphor Tyr31, ThermoFisher).

Generation of stable STAT3 reporter cells

MDA-MB-468 and MCF-7 were transfected with STAT3 reporter plasmid (Cignal Lenti STAT3 Reporter, QIAGEN, Hilden, Germany) using SureENTRY transduction reagent (Qiagen). Stable STAT3 reporting MDA-MB-468 and MCF-7 cells were selected with 10µg/ml and 8µg/ml of puromycin (Sigma) respectively over 10 days to generate stable STAT3 reporter MDA-468 and MCF-7 cell lines.

STAT3 Luciferase reporter assays

Stable STAT3 reporter MDA-MB-468 and MCF-7 breast cancer cells were seeded at 1 x 10^6 cells in a 100mm and cultured for 12 hrs and treated with 0.250µM niclosamide is added and incubated at 37°C, STAT3 promoter activity is measured at specific time points (0, 1, 6, 12, 24 and 48 hours). STAT3 promoter activity was determined by Promega Dual-Luciferase reporter assay system (Promega corporation, Madison, USA) following manufacturers instruction and luciferase activity measured in the Tecan™ microplate-Luminometer (Tecan Group limited,

Männedorf, Switzerland). The constitutively expressed non-inducible Renilla luciferase activity served as internal control for normalizing transfection efficiencies.

Statistical analysis

Data were analysed, and graphs plotted with GraphPad Prism version 6 software (GraphPad Inc.). Student's t-test was used to compare differences between two groups and multiple analysis was performed using analysis of variance (ANOVA). Multiple analysis of groups was checked for after ANOVA using Bonferroni's multiple comparison test. Statistical significance was defined as $P < 0.05$. For co-localization experiments, using ImageJ Pearson correlation coefficients were calculated. The Pearson's correlation coefficient reflects the degree of linear relationship between two fluorescence intensities. Co-localization coefficient >0.5 was defined as positive correlation, and 0.5 or less as no co-localization.

Extended Methods References

1 Lee, Y. H., Petkova, A. P., Mottillo, E. P. & Granneman, J. G. In vivo identification of bipotential adipocyte progenitors recruited by beta3-adrenoceptor activation and high-fat feeding. *Cell Metab* **15**, 480-491, doi:10.1016/j.cmet.2012.03.009 (2012).

2 Franken, N. A., Rodermond, H. M., Stap, J., Haveman, J. & van Bree, C. Clonogenic assay of cells in vitro. *Nat Protoc* **1**, 2315-2319, doi:10.1038/nprot.2006.339 (2006).

3 Kakuguchi, W. *et al.* HuR knockdown changes the oncogenic potential of oral cancer cells. *Mol Cancer Res* **8**, 520-528, doi:10.1158/1541-7786.MCR-09-0367 (2010).

4 Liu, Z. Q., Mahmood, T. & Yang, P. C. Western blot: technique, theory and trouble shooting. *N Am J Med Sci* **6**, 160, doi:10.4103/1947-2714.128482 (2014).
